# Expression and efficient secretion of a functional chitinase from *Chromobacterium violaceum* in *Escherichia coli*

**DOI:** 10.1186/1472-6750-13-46

**Published:** 2013-06-01

**Authors:** Marina Duarte Pinto Lobo, Fredy Davi Albuquerque Silva, Patrícia Gadelha de Castro Landim, Paloma Ribeiro da Cruz, Thaís Lima de Brito, Suelen Carneiro de Medeiros, José Tadeu Abreu Oliveira, Ilka Maria Vasconcelos, Humberto D’Muniz Pereira, Thalles Barbosa Grangeiro

**Affiliations:** 1 Departamento de Bioquímica e Biologia Molecular, Universidade Federal do Ceará, Fortaleza, CE CEP 60.455-970, Brazil; 2 Departamento de Biologia, UFC, Laboratório de Genética Molecular, Fortaleza, CE, Brazil; 3 Instituto de Física de São Carlos, Universidade de São Paulo, Av. Trabalhador Sãocarlense, 400, São Carlos, SP CEP 13.566-590, Brazil

**Keywords:** Signal peptide, Chitin-binding domain, Chitinase, Heterologous, Secretion

## Abstract

**Background:**

*Chromobacterium violaceum* is a free-living β-proteobacterium found in tropical and subtropical regions. The genomic sequencing of *C*. *violaceum* ATCC 12472 has revealed many genes that underpin its adaptability to diverse ecosystems. Moreover, *C*. *violaceum* genes with potential applications in industry, medicine and agriculture have also been identified, such as those encoding chitinases. However, none of the chitinase genes of the ATCC 12472 strain have been subjected to experimental validation. Chitinases (EC 3.2.1.14) hydrolyze the β-(1,4) linkages in chitin, an abundant biopolymer found in arthropods, mollusks and fungi. These enzymes are of great biotechnological interest as potential biocontrol agents against pests and pathogens. This work aimed to experimentally validate one of the chitinases from *C*. *violaceum*.

**Results:**

The open reading frame (ORF) CV2935 of *C*. *violaceum* ATCC 12472 encodes a protein (439 residues) that is composed of a signal peptide, a chitin-binding domain, a linker region, and a C-terminal catalytic domain belonging to family 18 of the glycoside hydrolases. The ORF was amplified by PCR and cloned into the expression vector pET303/CT-His. High levels of chitinolytic activity were detected in the cell-free culture supernatant of *E*. *coli* BL21(DE3) cells harboring the recombinant plasmid and induced with IPTG. The secreted recombinant protein was purified by affinity chromatography on a chitin matrix and showed an apparent molecular mass of 43.8 kDa, as estimated by denaturing polyacrylamide gel electrophoresis. N-terminal sequencing confirmed the proper removal of the native signal peptide during the secretion of the recombinant product. The enzyme was able to hydrolyze colloidal chitin and the synthetic substrates *p*-nitrophenyl-β-D-*N*,*N*’-diacetylchitobiose and *p*-nitrophenyl-β-D-*N*,*N*’,*N*”-triacetylchitotriose. The optimum pH for its activity was 5.0, and the enzyme retained ~32% of its activity when heated to 60°C for 30 min.

**Conclusions:**

A *C*. *violaceum* chitinase was expressed in *E*. *coli* and purified by affinity chromatography on a chitin matrix. The secretion of the recombinant protein into the culture medium was directed by its native signal peptide. The mature enzyme was able to hydrolyze colloidal chitin and synthetic substrates. This newly identified signal peptide is a promising secretion factor that should be further investigated in future studies, aiming to demonstrate its usefulness as an alternative tool for the extracellular production of recombinant proteins in *E*. *coli*.

## Background

Chitin is a linear homopolymer of β-(1,4)-linked *N*-acetyl-D-glucosamine (GlcNAc) residues and is the second most abundant organic compound in nature after cellulose [1]. This polysaccharide is an important structural component of the cell wall of many fungi and certain algae, the exoskeleton of insects and crustaceans, and the shell of mollusks and nematode eggs. It is also found in the peritrophic matrix, a chitin and glycoprotein layer that lines the midgut of most invertebrates [2].

Chitinases (EC 3.2.1.14) hydrolyze the β-(1,4)-linkages in chitin and chitodextrins, and they are present in organisms from all three domains of life. These enzymes play roles in a variety of processes, such as nutrition, parasitism, defense mechanisms and morphogenesis [3]. Based on the amino acid sequence similarities of their catalytic domains, chitinases are classified into families 18 and 19 of the glycoside hydrolases (GHs) [4]. GH18 chitinases are widespread in nature, occurring in viruses, archaea, bacteria, fungi, plants (classes III and V) and diverse groups of animals, such as insects and mammals [5]. Conversely, GH19 chitinases have a more restricted distribution, and to date, they have been found in higher plants (classes I, II and IV), nematodes, viruses and some groups of bacteria [6]. Furthermore, GH18 and GH19 chitinases have different catalytic mechanisms. GH18 members exert the retaining mechanism, in which the products of hydrolysis are β-anomers, whereas GH19 members exhibit the inverting mechanism, which produces α-anomers after catalysis [7-9]. Additionally, the chitinases of the two families do not share similarities in their amino acid sequences and display completely different three-dimensional structures, suggesting that they diverged from distinct ancestors [10]. However, both exo- and endochitinases are found in each family. Exochitinases release *N*,*N*’-diacetylchitobiose [(GlcNAc)_2_] or *N*,*N*’,*N*”-triacetylchitotriose [(GlcNAc)_3_] from the reducing or non-reducing end of the chitin chain in a processive manner. In contrast, endochitinases cleave chitin randomly at internal sites, producing low molecular mass oligomers that contain 2 to 6 units of GlcNAc [11].

Chitinases have attracted the interest of biotechnologists because of the many potential applications of these enzymes in medicine, agriculture and industry. For example, the antifungal properties of some bacterial and plant chitinases, which hydrolyze the cell walls of phytopathogenic fungi, have been intensively investigated as an alternative approach to protect crops against fungal diseases [12]. Moreover, it has been shown that *N*-acetyl chitooligosaccharides and GlcNAc, which can be produced from the hydrolysis of chitin by chitinases, possess interesting pharmacological properties that may be relevant for medical applications. *N*-acetyl chitooligosaccharides, for example, display antibacterial activity [13], while GlcNAc has been evaluated in clinical trials as a candidate to treat osteoarthritis and other joint disorders as well as inflammatory bowel disease [14].

*Chromobacterium violaceum* is a Gram-negative, facultative anaerobic β-proteobacterium (family Neisseriaceae) that is commonly found as a saprophyte in the water and soil in tropical and subtropical regions [15]. The complete genomic sequence of *C*. *violaceum* ATCC 12472 has been determined, revealing important molecular clues that underpin the versatility and adaptability of this free-living microorganism [16]. In addition to shedding light on particular aspects of the biology of *C*. *violaceum*, the analysis of its genome also has revealed many new genes with potential biotechnological applications in medicine, industry, environmental remediation and agriculture [16,17]. Of the *C*. *violaceum* genes that encode carbohydrate-degrading enzymes, several chitinase genes were identified in the genome of the strain ATCC 12472.

Therefore, the multiple chitinases unveiled by the genomic sequencing of *C*. *violaceum* ATCC 12472 may represent a new source of biocontrol molecules against phytopathogens and plant-parasitic nematodes. These chitinases may also have novel enzymatic and biological properties that would justify their future exploitation for practical purposes. However, none of the chitinases encoded by *C*. *violaceum* ATCC 12472 has been subjected to experimental validation to date.

The present work aimed to produce one of the chitinolytic enzymes of *C*. *violaceum* ATCC 12472 in *E*. *coli*. The recombinant protein was efficiently secreted into the culture medium via its native signal peptide and was purified to homogeneity by exploiting its ability to bind to insoluble chitin.

## Results

### Sequence analysis

**Figure 1:**
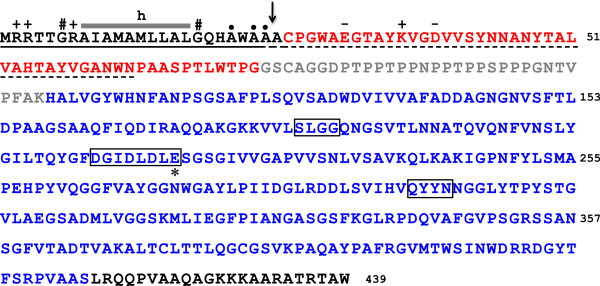
**Primary structure of the *Cv*Chi45 protein encoded by the CV2935 ORF of *C*. *violaceum *ATCC 12472.** The amino acid sequence of *Cv*Chi45 is shown, highlighting the N-terminal signal peptide (underlined with a continuous line), the ChBD (in red), the Pro/Thr-rich linker (in gray), the CatD (in blue) and the C-terminal extension (in black). The central h-region of the signal peptide (SP) is indicated by a gray bar. The symbols above the SP sequence refer to positively (+)-charged residues in the n-region (before the h-region), Gly residues (#) flanking the h-region, and Ala residues (●) in the c-region (after the h-region). The boundaries between these regions were determined by the SignalP 3.0 program [66]. The N-terminal sequence that was experimentally determined for the recombinant protein, which was purified from the culture medium of induced *E*. *coli* cells, is underlined with a dashed line. The actual SPase I cleavage site is indicated by an arrow. Positively (+)- and negatively (-)-charged residues within the first twenty N-terminal residues of the mature protein are also indicated. Structural motifs in the CatD that are involved in substrate binding and catalysis are boxed, and the crucial catalytic Glu residue is indicated by an asterisk (*). The numbers of the residues relative to Met1 are shown on the right side of the sequence.

The ORF CV2935 of *C*. *violaceum* ATCC 12472 encodes a putative GH18 chitinase [GenBank: AAQ60603] with 439 amino acid residues. The predicted pI and molecular mass of the encoded polypeptide chain are 8.9 and 45.7 kDa, respectively. The protein has a modular structure (Figure 1) and is composed of a 23-residue signal peptide at the N-terminus, a chitin-binding domain (ChBD; residues 26 to 74), followed by a Pro/Thr/Gly-rich linker region (residues 75 to 106), a catalytic domain (CatD; residues 107 to 416) at the C-terminal region, and a C-terminal extension (residues 417-439). The presence of the signal peptide suggests that the protein is synthesized as a pre-protein that is presumably targeted to the general secretory (Sec) pathway of *C*. *violaceum*. The protein is herein referred to as *Cv*Chi45 (for chitinase precursor of 45 kDa from *C*. *violaceum*).

**Figure 2:**
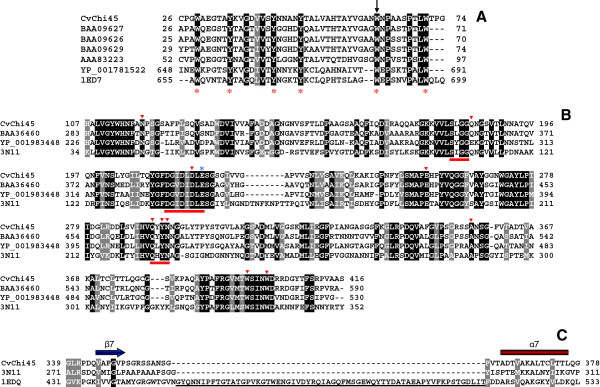
**Multiple sequence alignments of the ChBD and CatD of *Cv*Chi45. A**) Multiple sequence alignment of the ChBD of *Cv*Chi45 and related bacterial ChBDs. The sequences (retrieved from GenBank) are from *Aeromonas* sp. 10S-24 (accession numbers BAA09627, BAA09626, and BAA09629), *Janthinobacterium lividum* (AAA83223), *Clostridium botulinum* B1 str. Okra (YP_001781522), and *Bacillus circulans* WL-12 (PDB code: 1ED7). Conserved aromatic residues are indicated by red asterisks, and the Trp residue that plays a major role in ligand binding in the ChBD of *B*. *circulans* WL-12 ChiA1 is indicated by an arrow. **B**) Multiple sequence alignment of the CatD of *Cv*Chi45 and the CatDs from other bacteria (sequences were retrieved from GenBank): *Xanthomonas* sp. AK (BAA36460), *Cellvibrio japonicus* Ueda107 (YP_001983448), and *B*. *cereus* (3N11). Structural motifs involved in substrate binding and catalysis are indicated by red bars. Key residues that play a role in substrate binding and catalysis in the *B*. *cereus* chitinase are indicated by red triangles, whereas the crucial catalytic Glu residue is indicated by a blue asterisk. **C**) Alignment of a segment of the CatD_*Cv*Chi45_ and the corresponding region of the CatDs from the *B*. *cereus* (3N11) and *Serratia marcescens* (1EDQ) chitinases, respectively. The structural alignment between 3N11 and 1EDQ was obtained from the Dali database [70]. The β7 strand (blue arrow) and α7 helix (red rectangle) of the (β/α)_8_ barrel are indicated above the alignment, and the sequence of the chitin insertion domain (CID) in the ChiA of *S*. *marcescens* is underlined. Alignments were generated using ClustalW [69] and shaded as follows: positions with conserved residues are shaded in black, whereas those containing conservative substitutions are shaded in grey. In all alignments, the numbers of the residues relative to the Met1 of each protein are shown on the left and right sides of each sequence.

The chitin-binding domain of *Cv*Chi45 (ChBD_*Cv*Chi45_) is a member of the ChtBD type 3 (ChtBD3) superfamily and belongs to carbohydrate-binding module family 12 (CBM12) according to its CAZy classification. BLAST searches against the NCBI protein database detected high identity of the ChBD_*Cv*Chi45_ to diverse ChBDs of bacterial origin, such as those found in three chitinases from *Aeromonas* sp. 10S-24 (59.0%, 61.3% and 63.6% identities, respectively), the ChBD of a *Janthinobacterium lividum* chitinase (54.5% identity) and the ChBD of a carbohydrate-binding protein from the *Clostridium botulinum* B1 strain Okra (52.2% identity) (Figure 2A). The ChtBD3 superfamily includes modules of ~40-60 residues that bind cellulose and/or chitin. One feature of this ChBD is the presence of six conserved aromatic residues (corresponding to Trp^29^, Tyr^35^, Tyr^43^, Tyr^48^, Trp^62^, and Trp^71^ in the primary structure of *Cv*Chi45; Figure 2A) as well as three residues with hydrophobic side chains (Val^41^, Ala^50^, and Leu^70^ in the amino acid sequence of *Cv*Chi45) that are believed to be important in determining the domain structure and chitin binding ability (Figure 2A). For instance, the ChBD of *Bacillus circulans* WL-12 chitinase A1 (ChBD_ChiA1_) has a globular and compact structure with the topology of a twisted β-sandwich and contains two antiparallel β-sheets, which are composed of three and two strands, respectively, and a core region formed by the aromatic and hydrophobic residues [18]. The five aromatic (Trp^656^, Tyr^662^, Tyr^670^, Tyr^675^, and Trp^696^) and the three hydrophobic (Val^668^, Cys^677^, and Leu^695^) residues that contribute to the core region of ChBD_ChiA1_ are conserved in the ChBD_*Cv*Chi45_, except that Cys^677^ is replaced with Ala^50^ in the *Cv*Chi45. The sixth conserved aromatic residue in the ChBD_ChiaA1_, Trp^687^ (corresponding to Trp^62^ in the *Cv*Chi45 primary structure) is located on the surface of the protein where it has a major role in ligand binding, which is most likely mediated by hydrophobic stacking interactions with the pyranose rings of the substrate, as suggested by binding assays and mutagenesis data [19-21]. In ChiA1 of *B*. *circulans* WL-12 and in other bacterial chitinases, the non-catalytic ChBD is important for the interaction of the enzyme with insoluble chitin and is crucial for the efficient hydrolysis of chitin fibers by the catalytic domain [22,23]. Therefore, it is likely that in *Cv*Chi45, the N-terminal ChBD exerts a similar effect.

The ChBD_*Cv*Chi45_ is connected to the CatD by a 31-residue linker sequence that is rich in proline, threonine and glycine (Figure 1). In modular bacterial GHs, such as cellulases, xylanases, and chitinases, flexible disordered segments that are rich in proline and hydroxyamino acid residues (serine and threonine) as well as alanine and glycine, which are called PT-rich linkers, are commonly found to connect the non-catalytic substrate-binding domain to the enzymatic domain [24]. Experimental evidence suggests that these linkers form extended, flexible hinges, which determine the relative orientation of the binding and catalytic domains, thus optimizing the binding and catalytic efficiency of the enzyme on insoluble substrates [25].

The catalytic domain (CatD) of *Cv*Chi45 (CatD_*Cv*Chi45_) comprises *ca*. 2/3 of the encoded protein, in which the signature pattern of the active site of GH18 chitinases (consensus sequence: [LIVMFY]-[DN]-G-[LIVMF]-[DN]-[LIVMF]-[DN]-x-E; PROSITE accession number PS01095) corresponds to the segment ^212^FDGIDLDLE^220^. The three consensus motifs that are conserved in the catalytic domains of GH18 chitinases are found in the CatD_*Cv*Chi45_ (Figure 2B). These structural motifs are involved in substrate binding (^180^SxGG^183^) and catalysis (^213^DxxDxDxE^220^ and ^292^QXYN^295^; numbers refer to the *Cv*Chi45 amino acid sequence), respectively [9,10,26]. The catalytic domain of GH18 chitinases typically adopts a (β/α)_8_ triose-phosphate isomerase (TIM) barrel fold and uses a general acid/base, substrate-assisted double-displacement hydrolysis mechanism, leading to the retention of the configuration of the anomeric carbon [8,9]. The segment DxxDxDxE forms the β4 strand of the (β/α)_8_-barrel and constitutes the core of the catalytic center, in which the glutamic acid residue (Glu^220^ in the primary structure of *Cv*Chi45) is the crucial proton donor for catalysis [9,27]. The two aspartic acid residues (Asp^216^ and Asp^218^ in the *Cv*Chi45 sequence) that precede the catalytic Glu also play key roles in substrate hydrolysis. The aspartate nearest the catalytic Glu is referred to as the stabilizer, and its side-chain is believed to (i) orient the *N*-acetyl group of the GlcNAc residue bound to the −1 subsite, favoring the nucleophilic attack of the carbonyl oxygen on its anomeric center; (ii) stabilize the oxazolinium ion intermediate, and (iii) lower the p*K*_a_ of the acid/base glutamate. The second aspartate (defined as the stabilizer assistant) is suggested to raise the p*K*_a_ of the stabilizer residue [9,28-30]. The *Cv*Chi45 catalytic domain showed higher sequence identity to the GH18 domains of chitinases from *Xanthomonas* sp. AK (71.8% identity), *Cellvibrio japonicus* Ueda107 (62.5% identity) and *Chitiniphilus shinanonensis* (71.8% identity) (Figure 2B). Significant similarity (41.2% sequence identity) was also found between the CatD of *Cv*Chi45 and that of the *B*. *cereus* NCTU2 chitinase (ChiNCTU2), which contains a single catalytic domain without accessory domains and whose three dimensional structure has recently been resolved [31]. The 3D structures of wild-type and mutant ChiNCTU2 complexes with *N*-acetyl chitooligosaccharides have allowed for the identification of the key active site residues. The ChiNCTU2 residues that play crucial roles in substrate binding and catalysis are also conserved in the CatD_*Cv*Chi45_ sequence (Asn^118^, Gln^184^, Asp^218^, Glu^220^, Glu^257^, Gln^292^, Tyr^294^, Asn^295^, Ala^356^, Trp^397^, and Trp^401^) (Figure 2B). These sequence analyses suggest that the CV2935 ORF of *C*. *violaceum* ATCC 12472 likely encodes a functional GH18 chitinase that contains an accessory domain that is able to bind to chitin.

Furthermore, GH18 chitinases also differ in the presence or absence of a small (α + β)-fold domain (the chitin insertion domain, CID) within their CatDs. This domain is ~70-90 residues long and is inserted between the β7 strand and α7 helix of the (β/α)_8_ TIM-barrel structure of the CatD. The (α + β)-fold insertion domain is located at the top of the (β/α)_8_ TIM-barrel, creating a deep, tunnel-like substrate-binding cleft. In contrast, chitinases that do not contain this insertion domain have a shallow substrate-binding groove. This difference in the architecture of the substrate-binding groove has been correlated with the exo-type activity of CID-containing chitinases and the endo-type activity of chitinases without the CID [32]. Because the CatD_*Cv*Chi45_ does not have this insertion domain (Figure 2C), it is likely that this enzyme is an endochitinase. To validate these predictions, the protein encoded by the CV2935 ORF was produced using an *E*. *coli* expression system.

### Recombinant protein expression

An expression vector (pET-CV2935) containing the entire coding sequence of *Cv*Chi45 (including its native signal peptide) was obtained, and *E*. *coli* BL21(DE3) cells were transformed with this vector. The expression of the recombinant protein (r*Cv*Chi45) was achieved by adding IPTG to the culture medium. The levels of chitinolytic activity in the *E*. *coli* cells transformed with the recombinant plasmid were determined in a time-course experiment. The total chitinase activity against colloidal chitin, which was detected in soluble intracellular extracts from the transformed cells, was very low (86 U on average) even after 24 h of induction. Comparable background levels were also observed in intracellular extracts from control cells (*i*.*e*., *E*. *coli* harboring the expression vector pET303/CT-His without an insert) cultivated under the same conditions. However, high levels of chitinolytic activity (approximately 1,378.9 U after 24 h of induction) were detected in the cell-free culture supernatant of *E*. *coli* cells transformed with pET-CV2935 after IPTG induction. The chitinolytic activity in the extracellular fraction of the recombinant *E*. *coli* cells was detected as early as 2 h after induction and increased thereafter until 24 h. In contrast, chitinase activity was almost absent (approximately 3.5 U) in the cell-free culture supernatant of *E*. *coli* cells transformed with the empty vector and cultivated under the same experimental conditions (Additional file 1: Figure S1). These results suggest that the secretion of r*Cv*Chi45 by the *E*. *coli* cells was directed by its native signal peptide.

**Figure 3:**
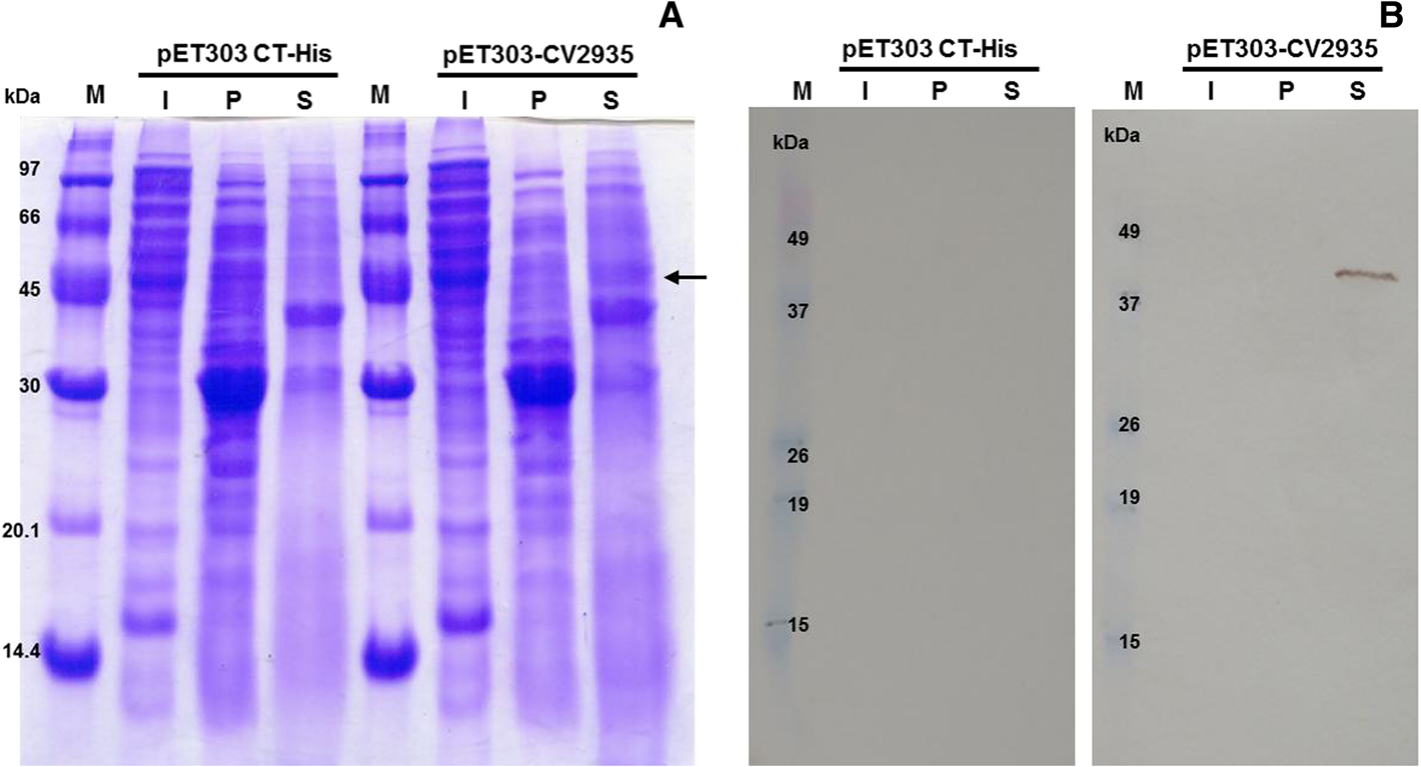
**Detection of *Cv*Chi45 in protein fractions of *E*. *coli *by SDS-PAGE (A) and Western blotting (B).** Protein fractions (soluble intracellular extract, I; periplasm extract, P; and cell-free medium supernatant, S) from *E*. *coli* cells transformed with pET303/CT-His or pET-CV2935 and induced under the same conditions were prepared as described in the Methods section. Proteins (100 μg per lane) were resolved by SDS-PAGE and stained with Coomassie Brilliant Blue (**A**) or transferred to a nitrocellulose membrane and submitted to immunodetection using an anti-His tag antibody (**B**), as described in the Methods section. The protein band corresponding to r*Cv*Chi45 is indicated by an arrow. M: molecular weight markers.

To determine the ability of the *E*. *coli* cells to secrete the recombinant chitinase, fractions from culture medium and cells were harvested at 24 h after induction, and analyzed by SDS-PAGE and Western blotting. Coomassie brilliant blue staining revealed a protein band with an apparent molecular mass of approximately 43.8 kDa that was evident only in the cell-free culture supernatant of *E*. *coli* cells transformed with pET-CV2935 and induced with IPTG (Figure 3A). This 43.8 kDa protein band reacted specifically with an anti-His6 tag antibody, and this reaction was not observed in the soluble intracellular extract and the periplasm fraction of *E*. *coli* cells containing pET-CV2935 (Figure 3B). The total chitinolytic activity was also measured in these fractions at 24 h after induction. The highest proportion (83.7%) of the hydrolytic activity was found in the growth medium, with minor amounts in the periplasmic fraction (9.4%) and the soluble cell lysate (6.9%). Therefore, approximately 93% of the soluble recombinant chitinase produced by the *E*. *coli* was exported, and the majority of the exported fraction present in the periplasmic space was secreted into the culture medium.

To evaluate if the level of recombinant chitinase secreted by the *E*. *coli* could be increased with the use of Terrific broth (TB), a growth medium that is richer than Lysogeny broth (LB) and that supports higher cell densities, a comparative time-course induction experiment was carried out. Higher levels of total soluble protein (approximately 1.4-fold) were secreted by the *E*. *coli* cells transformed with pET-CV2935 and cultivated in TB compared to transformed cells grown in LB medium and incubated under the same conditions. However, the activity of the chitinase secreted by the transformed cells cultivated in TB was lower (approximately 3-fold less at 24 h after induction) than that in the extracellular fraction of cells grown in LB (Additional file 2: Figure S2). In both conditions, the highest protein concentration and chitinolytic activity in the cell-free medium were observed 24 h after induction, and these levels decreased thereafter. We next aimed to purify the recombinant protein secreted into the culture medium using induced *E*. *coli* cells grown in LB for 24 h.

### Purification and characterization

**Figure 4:**
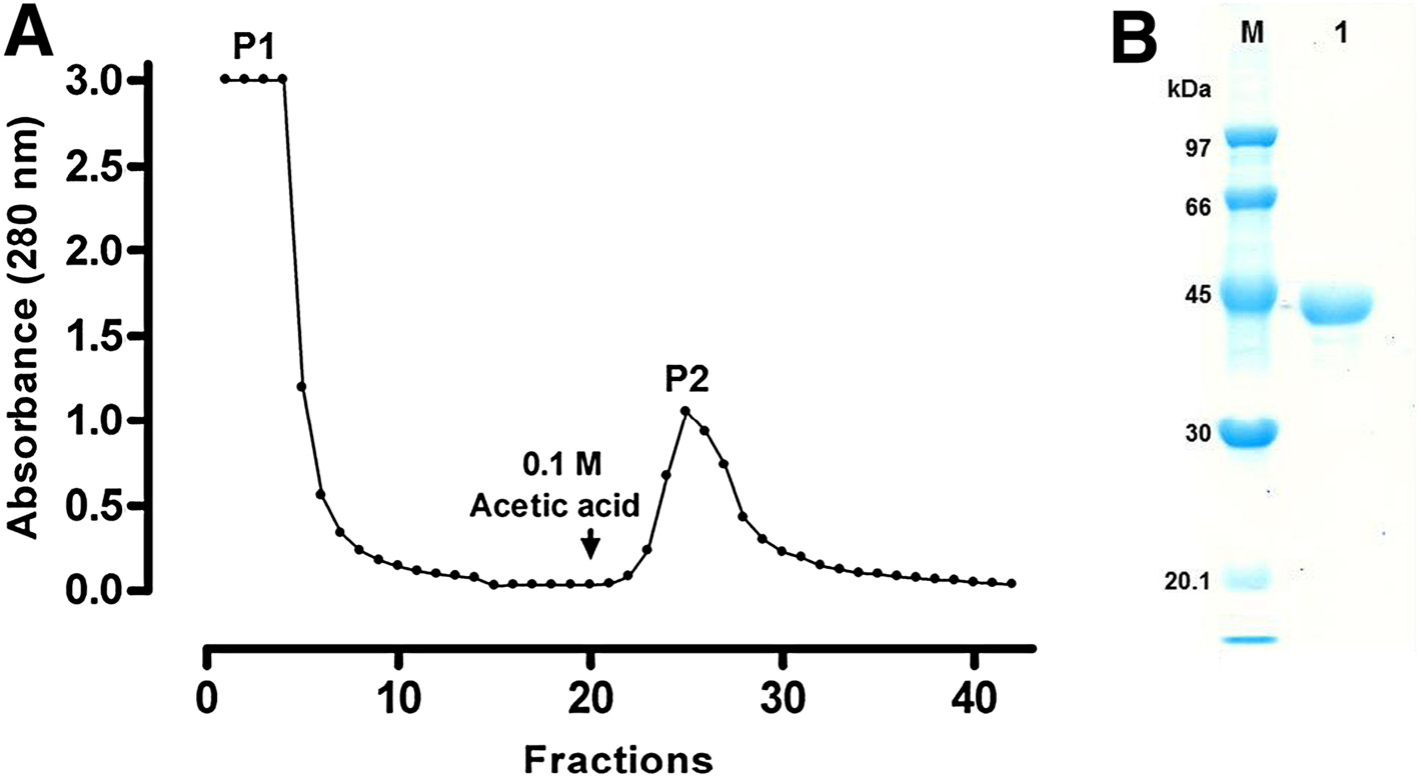
**Affinity chromatography on a chitin matrix (A) and SDS-PAGE analysis (B).** (**A**) Purification of recombinant *Cv*Chi45 secreted into the *E*. *coli* culture medium. The secreted proteins were concentrated as described in the Methods section and loaded onto a chitin column that was equilibrated with NaAc buffer (pH 5.2) containing 1 M NaCl. After washing off the unbound proteins (P1), the recombinant protein was eluted with 0.1 M acetic acid (P2). (**B**) SDS-PAGE analysis of purified recombinant *Cv*Chi45 (lane 1). M: molecular weight markers.

To purify the *C*. *violaceum* chitinase produced in *E*. *coli*, the secreted recombinant protein was recovered from the cell-free culture supernatant by ammonium sulfate precipitation and further purified by single-step affinity chromatography on a chitin matrix. After washing out the unbound proteins (Peak 1 - P1) with equilibration buffer (Figure 4A), pure recombinant chitinase was eluted (Peak 2 - P2) by washing the column with 0.1 M acetic acid, as shown by SDS-PAGE (Figure 4B). A small amount of chitinolytic activity was detected in the pooled fractions of P1, but this value was approximately 1% of the total activity in the pooled fractions of P2, showing that the binding of r*Cv*Chi45 to the chitin matrix was effective. A purification factor of 14.9-fold was obtained, and *ca*. 60% of the total chitinolytic activity which was present in the cell-free culture supernatant was recovered. The yield of purified recombinant protein was *ca*. 4 mg per liter of induced culture, which represents approximately 7% of the total protein content in the concentrated cell-free culture medium. When submitted to electrophoresis under denaturing conditions (SDS-PAGE), the purified protein migrated as a single band with an apparent molecular mass of approximately 43.8 kDa, instead of 45.7 kDa, the expected size of the 439-residue precursor encoded by the DNA sequence cloned into the expression vector. However, taking into account the extra residues at the C-terminus (LEHHHHHH; molecular mass = 1,082.49 Da) corresponding to the His-tag sequence added to the recombinant protein, this value (43.8 kDa) is in good agreement with the predicted molecular mass (*ca*. 44.2 kDa) calculated for the expressed His-tagged fusion protein without its native signal peptide. The N-terminal sequence of the purified recombinant chitinase secreted into the culture medium was determined to be: ACPGEWAEGTAYKVGDVVSYNNANYTALVAHTAYVGANWN. This sequence corresponds to a 40-residue segment of the pre-protein, from Ala^25^ to Asn^63^, excluding the first 24 residues, as highlighted in Figure 1. The presence of a signal peptide at the N-terminal region of the protein sequence encoded by ORF CV2935 was predicted by the SignalP software (probability = 1.0, calculated using the hidden Markov model of SignalP). Furthermore, three most likely cleavage sites for type I signal peptidase (SPase I) were predicted within the first 70 residues in the precursor sequence of *Cv*Chi45. These sites were located between Ala^21^-Trp^22^ (cleavage site score = 0.288; neural network model of the SignalP algorithm), Ala^23^-Ala^24^ (C-score = 0.546) and Ala^24^-Ala^25^ (C-score = 0.360). As shown by the experimentally determined amino terminal sequence, the native signal peptide was cleaved between Ala^24^-Ala^25^, which was the second most likely SPase I cleavage site as predicted by the neural network model of the SignalP algorithm. Thus, the native signal peptide in the precursor sequence of *Cv*Chi45 was recognized by the general secretory apparatus of the *E*. *coli* and directed the secretion of the expressed protein into the culture medium. Moreover, the signal peptide of *Cv*Chi45 was correctly processed to produce a mature extracellular enzyme with chitinolytic and chitin-binding activities.

**Figure 5:**
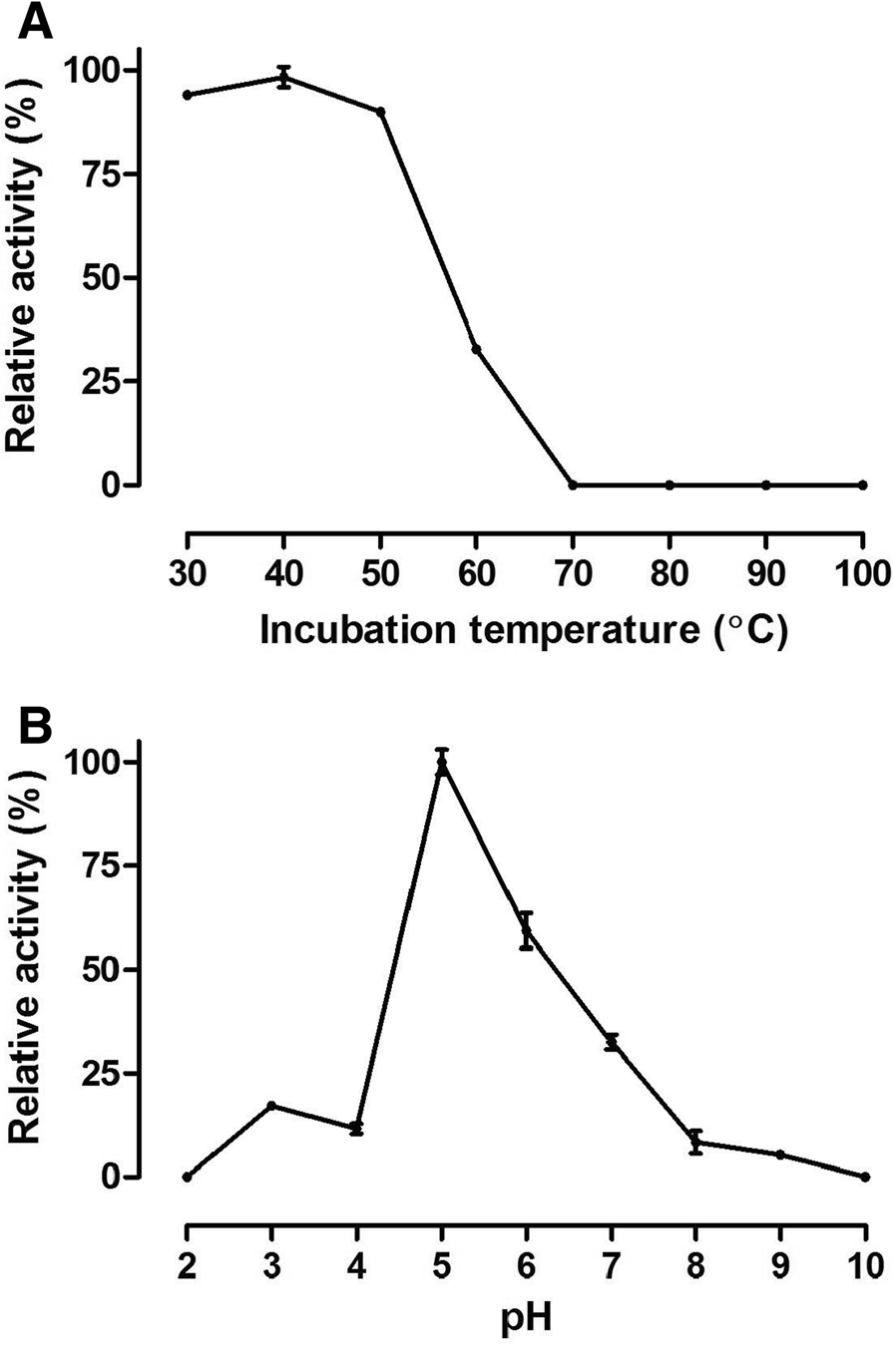
**Temperature stability (A) and pH activity (B) profiles of *Cv*Chi45.** (**A**) The recombinant protein (150 ng/μL) was incubated for 30 min at varying temperatures and then centrifuged (10,000 *g*, 10 min, 4°C), and the residual chitinolytic activity was determined in the supernatant as described in the methods section, using colloidal chitin as a substrate. (**B**) Samples of recombinant *Cv*Chi45 (150 ng/μL) were dialyzed for 1 h against buffers with different pH values, and the hydrolytic activity was determined under standard assay conditions using colloidal chitin as a substrate. The relative activity was expressed as a percentage of the highest activity recorded at a certain temperature or pH, respectively.

To further characterize the recombinant chitinase, the effects of temperature, pH, and metal ions on its hydrolytic activity were evaluated. The purified enzyme was relatively thermostable, retaining approximately 32% of its activity after being heated to 60°C for 30 min. Enzymatic activity was completely lost when the incubation was carried out at 70°C or higher for 30 min (Figure 5A). The chitinolytic activity of the purified *Cv*Chi45 was detected over a wide pH range (3.0 to 9.0), but there was no activity detected at pH 2.0 and 10.0; the maximum relative activity was recorded at pH 5.0, with significant hydrolysis of colloidal chitin also detected at pH 6.0 (61.1%) and 7.0 (33.6%) (Figure 5B). Most of the tested metal ions (Ba^2+^, Ca^2+^, Cu^2+^, K^+^, Mg^2+^, Ni^2+^ and Zn^2+^) as well as the monovalent cation NH_4_^+^ did not affect the hydrolytic activity of *Cv*Chi45 against *p*NP-(GlcNAc)_2_ and *p*NP-(GlcNAc)_3_ at a concentration of 5 mM. However, Mn^2+^ and Fe^2+^ were effective in reducing the hydrolytic activity against both *p*NP-(GlcNAc)_2_ (30.3 and 74.6% inhibition, respectively) and *p*NP-(GlcNAc)_3_ (26.6 and 67.2% inhibition, respectively) (Additional file 3: Figure S3). There was no inhibitory effect of 5 mM EDTA or 5% β-mercaptoethanol on the enzymatic activity of *Cv*Chi45 against the two chromogenic *N*-acetyl-chitooligosaccharide analogues. In contrast, 0.5% SDS completely abolished the ability of the enzyme to hydrolyze *p*NP-(GlcNAc)_2_ and *p*NP-(GlcNAc)_3_.

The substrate specificity of *Cv*Chi45 was investigated by colorimetric assays using colloidal chitin and synthetic analogues (*p*NP-derivatives) of *N*-acetyl-chitooligosaccharides as substrates (Table 1). In the enzymatic assay using colloidal chitin as a substrate, free GlcNAc monomers were detected only when *Helix pomatia* β-glucuronidase was added to the reaction mixture, after the initial incubation of the enzyme sample with the substrate. Thus, the hydrolytic activity of *Cv*Chi45 on chitin chains liberated water-soluble chitin oligomers, which were converted to free GlcNAc by the β-glucuronidase. This result also reveals the lack of β-*N*-acetylglucosaminidase (GlcNAcase) activity (EC 3.2.1.52). GlcNAcases remove β-linked GlcNAc from the non-reducing end of different substrates including oligosaccharides, for example. These enzymes are classified into families GH3, GH20 and GH84, and although some of them are involved in the degradation of chitin, they are not chitinases [33]. The substrate specificity of r*Cv*Chi45 was further examined using chromogenic analogues of di- (*p*NP-GlcNAc), tri- [*p*NP-(GlcNAc)_2_] and tetra-oligomers [*p*NP-(GlcNAc)_3_] of GlcNAc as substrates. There was no detectable activity against *p*NP-GlcNAc, indicating an absence of GlcNAcase activity, which is consistent with the result found in the assay using colloidal chitin as a substrate. On the other hand, high specific activities were found against *p*NP-(GlcNAc)_2_ and *p*NP-(GlcNAc)_3_, respectively. The recombinant enzyme exhibited almost the same specific activity against both of these substrates. The results suggest that *Cv*Chi45 cleaves internal *O*-glycosidic linkages, thus removing the *N*,*N*’-diacetylchitobiose and *N*,*N*’-,*N*”-triacetychitotriose moieties from the non-reducing end of *N*-acetyl chitooligomers and chitin.

**Table 1:** **Specific activity (U/mg) against colloidal chitin and synthetic substrates of *Cv*Chi45**

**Substrate**	***Cv*Chi45**	***S. griseus *chitinase**
Colloidal chitin*	22,260.5	20,368.0
*p*-nitrophenyl-*N*-acetyl-β-D-glucosamine (*p*NP-GlcNAc)**	ND	ND
*p*-nitrophenyl-β-D-*N*,*N*’-diacetylchitobiose [*p*NP-(GlcNAc)_2_]**	32,320.0	10,370.0
*p*-nitrophenyl-β-D-*N*,*N*’,*N*”-triacetylchitotriose [*p*NP-(GlcNAc)_3_]**	31,560.0	9,120.0

The specific activity (U/mg) of the purified chitinase was measured using colloidal chitin and synthetic substrates as described in the Methods section. A chitinase from *Streptomyces griseus* (Sigma) was included for comparison.

* For assays using colloidal chitin as the substrate (0.5% final concentration), one unit was defined as the amount of enzyme that released 1 nmol of GlcNAc/mg/h at 37°C.

** For assays using synthetic substrates, one unit was defined as the amount of enzyme that released 1 nmol of 4-nitrophenol/mg/h at 37°C; the substrates were assayed at final concentrations of 2.9 mM (*p*NP-GlcNAc), 1.8 mM [*p*NP-(GlcNAc)_2_], and 1.3 mM [*p*NP-(GlcNAc)_3_]; ND: not detected.

## Discussion

Large amounts of chitin are produced in nature by living organisms, especially by fungi and invertebrates, such as insects, crustaceans and mollusks. It has been estimated that more than 10^11^ metric tons of this polysaccharide are generated annually only in the aquatic biosphere [34]. Chitinolytic bacteria play a crucial role in recycling the chitinous structures that are continuously produced in various ecosystems [35]. To degrade chitin, bacteria secrete chitinases that hydrolyze the polysaccharide to soluble oligosaccharides, mostly *N*,*N*’-diacetylchitobiose. These oligosaccharides are then metabolized by GlcNAcases to yield the monosaccharide GlcNAc. *Chromobacterium violaceum* is a saprophyte bacterium found in the soil and water and is able to use chitin as the sole source of carbon and nitrogen [36]. *C*. *violaceum* produces multiple extracellular chitinases that are involved in this physiological process [37].

In this work, a GH18 chitinase (*Cv*Chi45) encoded in the genome of *C*. *violaceum* ATCC 12472 was produced and efficiently secreted into the culture medium of *E*. *coli* BL21(DE3) cells. The *C*. *violaceum* chitinase was directed to the extracellular medium by its native signal peptide (SP_*Cv*Chi45_), which was properly removed during the secretion of the pre-protein. Translocation across the inner cell membrane of *E*. *coli* has been reported for chitinases from diverse bacteria such as *Aeromonas caviae*[38], *A*. *hydrophila*[39], *Alteromonas* sp. strain O-7 [40], *B*. *cereus*[41], *B*. *circulans*[42], *Enterobacter agglomerans*[43], *Janthinobacterium lividum*[44], *S*. *marcescens*[45], *Streptomyces plicatus*[46], and *Vibrio parahaemolyticus*[47]. In all these studies, the translocation of the enzymes across the inner cell membrane of *E*. *coli* was mediated by the native signal peptides. Once exported by *E*. *coli*, some of these chitinases remained in the periplasm [40,42,44,46] but others were secreted into the culture medium [39,41,43,45,47].

One can speculate that in strain ATCC 12472, the *C*. *violaceum* chitinase encoded by ORF CV2935 is most likely involved in the first hydrolytic reactions that lead to the depolymerization of chitin. Another plausible hypothesis is that once secreted, this *C*. *violaceum* chitinase might also have an antagonistic activity on fungi and nematode eggs, as reported for some soil strains of *Chromobacterium*[48,49].

The recombinant product *Cv*Chi45 was purified to homogeneity by a single-step procedure that employed affinity chromatography on a chitin matrix. The interaction between the recombinant chitinase and the matrix of insoluble chitin was likely mediated by the ChBD present in *Cv*Chi45. The purified recombinant protein displayed highest chitinolytic activity at pH 5.0, and this characteristic may have physiological significance because *C*. *violaceum* and other *Chromobacterium* species are known to thrive in aquatic and terrestrial sites with acidic pH [50]. The acidic pH-optimum of GH18 chitinases like *Cv*Chi45, hevamine [51] and Ech30 (from *Trichoderma atroviride*) [52] is probably due to a conserved Asn residue (Asn^295^ in the amino acid sequence of *Cv*Chi45, as highlighted in Figure 2B) near the catalytic Glu. ChiB from *S*. *marcescens*, which contains an Asp at this position (Asp^215^), has a broad pH-activity profile and shows highest activity at neutral pH. Substitution of Asp^215^ for Asn yielded an acidic ChiB mutant, with very low activity at pH 7, but significant (wild-type like) activity at pH 4.5 [9].

The enzymatic activity of *Cv*Chi45 was not inhibited by the metal ion-chelating compound EDTA or the reducing agent β-mercaptoethanol, suggesting that metal ions and disulfide bonds may not be important in the stability and/or activity of *Cv*Chi45. On the other hand, the divalent metal ions Mn^2+^ and Fe^2+^ caused significant reductions in the hydrolytic activity of *Cv*Chi45. This inhibitory effect probably involves the interaction of these divalent cations with the negatively-charged carboxylate residues in the chitinase active center. This assumption is based on previous observations that divalent metal ions are able to form stable complexes with carboxylic groups at the active sites of enzymes, as investigated in hen egg-white lysozyme [53]. Altogether, these results confirm the functional predictions that are based on sequence comparisons between the primary structures of *Cv*Chi45 and previously characterized bacterial chitinases.

The sequence of the signal peptide of *Cv*Chi45 is 24 residues long, and the N-, H- and C-regions are 6, 10, and 7 residues in length, respectively. Three Arg residues are found in the N-region of SP_*Cv*Chi45_, giving it a net positive charge of +3. In the Sec-dependent type I SPs of *E*. *coli* (SPI_*Ec*_), these positively charged residues maintain electrostatic interactions with the negatively charged phospholipids of the inner membrane [54,55]. The basic residues in the N-region of SPI_*Ec*_ also promote the association of the preprotein-secB complex with SecA [56-58]. In the H-region of SP_*Cv*Chi45_, Ala (4 residues) and Leu (3 residues) occur almost in the same proportion and are the most frequent residues, a profile that is similar to that found in SPI_*Ec*_. The stretch of hydrophobic residues in the H-region of SPI_*Ec*_ binds to a hydrophobic peptide-binding groove in the SecA structure and adopts an α-helical conformation in the inner membrane [59,60]. Two α-helix-breaking Gly residues (at positions -7 and -19, respectively) flank the H-region of SP_*Cv*Chi45_, one residue on each side of the central hydrophobic core. The Gly residue at the -7 position defines the border between the H- and C-regions. A similar profile is observed in the boundary between the H-region and the C-region of SPI_*Ec*_, where an α-helix-destabilizing residue (Pro or Gly) often occurs at or around the -6 position [61]. The C-region of SP_*Cv*Chi45_ has a predominance of Ala residues and the -1 and -3 positions (relative to the SPase I cleavage site) are occupied by Ala and Trp residues, respectively. In comparison, these positions in the C-region of SPI_*Ec*_ are occupied by residues with small aliphatic or polar side-chains. Because alanine is most often found at the -1 and -3 positions, the SPase I cleavage site specificity is usually referred to as the Ala-X-Ala rule [61]. Moreover, the first 18 residues of the mature protein *Cv*Chi45 have a negative net charge (Figure 1), a feature that has been shown to be important for protein secretion in *E*. *coli* and other Gram-negative bacteria [62]. Thus, SP_*Cv*Chi45_ is a type I SP that directs the export or secretion of proteins through the Sec pathway and is removed by signal peptidase I. Because SP_*Cv*Chi45_ possesses the major features of a prototypical *E*. *coli* type I SP, one can suggest that this concurrence of physico-chemical properties underlies the efficient secretion of the *C*. *violaceum* chitinase by *E*. *coli*. Once it has been folded in the *E*. *coli* periplasm, the recombinant protein most likely reaches the culture medium by nonspecific periplasmic leakage or through the second step of a type II secretion system [63].

High expression levels of foreign proteins in *E*. *coli* often lead to their accumulation in intracellular inclusion bodies. As a consequence, *in vitro* refolding of these insoluble proteins is necessary to restore their biological activity. One strategy to overcome this problem is the secretion of the heterologous protein into the culture medium. Some advantages of this approach include: more simple purification schemes, enhanced biological activity, higher stability and solubility of the expressed protein, and N-terminal authenticity of the recombinant product [63]. Among the signal sequences that have been used to secrete recombinant proteins in *E*. *coli*, we can cite, for example, those from the proteins OmpA, PhoA, SpA (protein A from *Staphylococcus aureus*), and pelB (pectate lyase B from *Erwinia carotovora*) [64]. However, the discovery of new signal peptides that are functional in *E*. *coli* is still a relevant matter. First, the efficiency of a signal sequence to direct the secretion of a target protein cannot be anticipated from the analysis of the properties of its sequence alone. Second, there is no guarantee that a signal sequence that is suitable for the secretion of a given protein will have the same efficiency to direct the secretion of a different one. Therefore, several signal peptides must be evaluated through a trial-and-error approach.

## Conclusions

In the present study, the functionality of a GH18 chitinase (*Cv*Chi45) that is encoded in the genome of *C*. *violaceum* ATCC 12472 was experimentally demonstrated using heterologous expression in *E*. *coli* BL21(DE3) cells. The native signal peptide allowed for the secretion of the recombinant product into the culture medium to a level of 4 mg/L in shake flask cultures. The signal sequence was correctly removed during secretion, and the mature protein in the extracellular medium was soluble, bound insoluble chitin and hydrolyzed colloidal chitin and synthetic analogues of *N*-acetyl chitooligosaccharides. The chitinase was purified to electrophoretic homogeneity and showed an apparent molecular mass of 43.8 kDa. The protein *Cv*Chi45 possesses promising functional domains that should be further studied, aiming for their exploitation as molecular tools for the heterologous expression in *E*. *coli* of foreign proteins with biotechnological applications.

## Methods

### Plasmid, bacterial strain and culture media

The plasmid pET303/CT-His and the *Escherichia coli* strain BL21(DE3) were purchased from Invitrogen (Carlsbad, CA, USA). The bacterial cells were cultivated in LB medium (10 g/L peptone, 5 g/L yeast extract, 5 g/L NaCl, pH 7.0). The effect of Terrific broth (TB; 12 g/L tryptone, 24 g/L yeast extract, 4 mL/L glycerol, 2.31 g/L KH_2_PO_4_ and 12.54 g/L K_2_HPO_4_) on the level of recombinant protein expression was also verified.

### Sequence analysis

Searches for homologous proteins on public sequence databases were performed using BLASTp [65]. Signal peptide and signal peptidase cleavage sites were predicted using SignalP version 3.0 [66]. The theoretical isoelectric point (pI) and molecular weight (Mw) were predicted using Compute pI/Mw on the ExPASy Proteomics Server [67]. The presence and delimitation of protein domains was accomplished using the Conserved Domain Database (CDD) [68]. Multiple amino acid sequence alignments were generated using ClustalW [69]. The identity between a pair of aligned sequences was calculated as the number of identical residues divided by the number of aligned positions, excluding the sites with gaps, and expressed as a percentage. Structural alignments based on comparisons of 3D structures deposited in the Protein Data Bank (PDB) were obtained from the Dali database [70]. The functional domains were named following the nomenclature adopted by the Carbohydrate-Active enZymes (CAZy) database [71].

### Amplification and cloning

Genomic DNA from *C*. *violaceum* ATCC 12472 was isolated using a CTAB-based protocol as described previously [72]. The complete DNA sequence of the CV2935 ORF was amplified by PCR using the bacterial genomic DNA as a template. The design of the PCR primers was based on the coding sequence of the corresponding ORF, which spans the *C*. *violaceum* chromosome from position 3,211,716 to 3,213,035 [GenBank accession number: NC_005085]. The primer sequences were 5’-CCGTCTAGAATGCGCAGAACGACAGGCAGG-3’ (forward) and 5’-CCGCTCGAGCCAGGCCGTCCGCGTCGCGCG-3’ (reverse). Restriction endonuclease sites (underlined) were incorporated in the forward (*Xba*I) and reverse (*Xho*I) primers, respectively, to allow for further manipulation of the PCR products. Amplifications were carried out in a final volume of 20 μL containing 50 ng genomic DNA, 1X Buffer Phusion GC (Finnzymes, Vantaa, Finland), 1.5 mM MgCl_2_, 200 μM of each dNTP, 0.5 μM of each primer, and 0.4 U Phusion Hot Start High-Fidelity DNA Polymerase (Finnzymes). Amplification reactions were performed in a Mastercycler Gradient Thermo Cycler (Eppendorf, Hamburg, Germany) using the following cycling parameters: an initial denaturation step (3 min at 98°C) followed by 35 cycles of 10 s at 98°C, 30 s at 65°C, and 2.5 min at 72°C. After the last cycle, the reactions were further incubated for 5 min at 72°C. The amplified product was verified by analyzing a 5-μL aliquot of the PCR reactions by 1.0% agarose gel electrophoresis [73]. PCR products were purified from the remaining solution using the illustra GFX PCR DNA and Gel Band Purification Kit (GE Healthcare, Buckinghamshire, UK), digested with *Xba*I and *Xho*I (Fermentas Life Sciences, Ontario, Canada) and purified again using the same kit. The PCR products were then ligated into the pET303/CT-His vector, which was previously digested with the same restriction enzymes, using T4 DNA Ligase (Promega, Madison, WI, USA). The ligation products were introduced into *E*. *coli* BL21(DE3) cells by electroporation, and the transformants were selected on LB agar containing 100 μg/mL carbenicillin. Plasmid DNA was isolated from antibiotic-resistant colonies using the alkaline lysis method [73], and the presence of the insert was confirmed by PCR and restriction digestion with the appropriate endonucleases. In the recombinant plasmid, designated pET-CV2935, the chitinase coding sequence was cloned in frame with a C-terminal polyhistidine (6xHis) tag and its expression was under the control of the T7*lac* promoter.

### Protein expression and purification

A single isolated colony of *E*. *coli* BL21(DE3) cells harboring the recombinant plasmid pET-CV2935, which were grown on LB agar supplemented with 100 μg/mL carbenicillin, was selected and inoculated in 5 mL LB containing the same antibiotic. The culture was incubated with vigorous shaking (180 rpm) at 37°C for 16 h. An aliquot (500 μL) of this culture was then inoculated into 50 mL LB (in a 500 mL Erlenmeyer flask) supplemented with 100 μg/mL carbenicillin and further incubated as before until the OD_600_ reached 0.4-0.5. Isopropyl-β-D-thiogalactopyranoside (IPTG) was added to a final concentration of 0.5 mM to induce the expression of the recombinant protein. After 24 h at 37°C, the culture was centrifuged (6,000 *g*, 10 min, 4°C), the cell pellet was saved for further analysis and the supernatant (*i*.*e*., the medium free of cells) was dialyzed against distilled water. To the dialyzed supernatant, solid ammonium sulfate was added to 95% saturation, and the mixture was incubated at room temperature for approximately 20 h. The precipitated proteins (F0/95) were collected by centrifugation (15,000 *g*, 20 min, 4°C) and resuspended in 50 mM sodium acetate (NaAc) buffer (pH 5.2) containing 1 M NaCl. The mixture was incubated for 3 h at 4°C, and the insoluble materials were then removed by centrifugation (15,000 *g*, 20 min, 4°C). The clear supernatant was loaded onto a chitin column (1.5 × 16 cm), which was prepared from practical grade chitin from crab shells (Sigma-Aldrich, St. Louis, MO, USA). The chitin was equilibrated with 50 mM NaAc buffer (pH 5.2) containing 1 M NaCl, and the protein sample was loaded onto the matrix and incubated for 16 h at 4°C. Unbound proteins were eluted by washing the column with the equilibration buffer, and the adsorbed proteins were recovered by elution with 0.1 M acetic acid. Fractions with an A_280_ greater than 0.050 were pooled and dialyzed against distilled water. The dialyzed material was centrifuged (12,000 *g*, 10 min, 4°C) and concentrated by ultrafiltration using a 30-kDa cut-off membrane (Vivaspin 20, GE Healthcare).

### Osmotic shock and cell lysis

Periplasmic proteins expressed in *E*. *coli* cells harboring the plasmid pET-CV2935 were obtained by the osmotic shock (OS) protocol described by Koshland and Botstein [74], with minor modifications. Briefly, the cells were resuspended in OS solution 1 [20 mM Tris-HCl (pH 8.0), 2.5 mM EDTA, 20% (w/v) sucrose] to an OD_550_ = 5.0, and the mixture was incubated on ice for 10 min and centrifuged (6,000 *g*, 10 min, 4°C). The buffer was decanted, and the cells were resuspended in OS solution 2 [20 mM Tris-HCl (pH 8.0), 2.5 mM EDTA] using the same volume as before. The cell suspension was again incubated on ice for 10 min and centrifuged (6,000 *g*, 10 min, 4°C), and the supernatant (shock fluid fraction) was transferred to a clean tube and stored at −20°C. The remaining cells were lysed by resuspending them in lysis buffer (50 mM Tris-HCl, pH 8.0, 2 mM EDTA, 0.15 M NaCl) containing 100 μg/mL lysozyme and 0.1% Triton X-100 (v/v). The suspension was incubated at 30°C for 30 min in a water bath, and the bacterial genomic DNA was then digested at 30°C for 30 min using DNase I (Promega) in the presence of 8 mM MgCl_2_ and 10 mM CaCl_2_. The lysate was centrifuged (12,000 *g* for 30 min at 4°C) and the supernatant, which was saved as the soluble intracellular extract, was kept at −20°C until use.

### Chitinolytic activity assays

Chitinolytic activity was determined by the colorimetric method described by Boller [75] using colloidal chitin as a substrate. Colloidal chitin was prepared according to Molano et al. [76] using non-radioactive acetic anhydride. In this assay, the hydrolytic activity of the chitinase releases water-soluble oligomers from colloidal chitin, and in a second reaction, these oligosaccharides are cleaved to GlcNAc by a β-glucuronidase [77]. The protein sample [250 μL, diluted in 50 mM NaAc buffer (pH 5.2)] was added to 250 μL of 10 g/L colloidal chitin, and the mixture was incubated at 37°C for 1 h. The reaction was quenched by boiling for 5 min in a water bath and then centrifuged (10,000 *g*, 25°C, 15 min), and 300 μL of the supernatant was transferred to a clean microcentrifuge tube containing 10 μL assay buffer [50 mM NaAc buffer (pH 5.2)] or 10 μL of 10 U/mL β-glucuronidase (EC 3.2.1.31) type HP-2 (Sigma-Aldrich). Reactions were further incubated at 37°C for 1 h, boiled for 5 min and cooled on ice. Assay buffer (190 μL) and 100 μL 0.6 M potassium tetraborate were added, and the reaction mixture boiled again for 5 min and cooled on ice for 3 min. Next, 1.0 mL of 5% (w/v) *p*-dimethylaminobenzaldehyde (DMAB, Sigma-Aldrich) prepared in 0.7 M HCl (diluted in 100% acetic acid) was added, and the absorbance at 585 nm was determined. The amount of GlcNAc released was estimated from a standard curve prepared with varying concentrations (100-600 μM) of GlcNAc [78]. Chitinase activity was expressed in units (U), and 1 U was defined as the amount of enzyme that released 1 nmol of GlcNAc/mL/h at 37°C. Substrate specificity was also investigated using chromogenic analogues of *N*-acetyl chitooligossacharides as substrates. The following synthetic *p*-nitrophenyl-labeled oligomers of GlcNAc were used: *p*-nitrophenyl-*N*-acetyl-β-D-glucosamine (*p*NP-GlcNAc), *p*-nitrophenyl-β-D-*N*,*N*’-diacetylchitobiose [*p*NP-(GlcNAc)_2_] and *p*-nitrophenyl-β-D-*N*,*N*’-,*N*”-triacetylchitotriose [*p*NP-(GlcNAc)_3_] (Sigma-Aldrich). Hydrolytic activity was determined by measuring the release of *p*NP from the substrates [79] according to the procedure supplied by the compounds’ manufacturer. The purified chitinase (10 μL; 150 ng protein/μL distilled water) was mixed with 90 μL of 1 mg/mL substrate solution, and the mixture was incubated at 37°C for 30 min. The reaction was quenched by adding 200 μL of 0.4 M sodium carbonate, and the A_405_ of the *p*-nitrophenylate ion was measured. One unit of hydrolytic activity was defined as the amount of enzyme releasing 1 nmol of *p*-nitrophenol/mL/h at 37°C.

### Soluble protein content

The concentration of soluble protein in the bacterial culture samples and the fractions obtained during protein purification was determined using the Bradford method [80] with bovine serum albumin as a standard.

### SDS-polyacrylamide gel electrophoresis and Western blotting

Polyacrylamide gel electrophoresis in the presence of sodium dodecyl sulfate (SDS) and β-mercaptoethanol (SDS-PAGE) was performed as described by Laemmli [81] using 15% slab gels. Samples were prepared in 0.0625 M Tris-HCl (pH 6.8) containing 2% (w/v) SDS, 5% (v/v) β-mercaptoethanol, 10% (v/v) glycerol and 0.001% (w/v) bromophenol blue. Protein bands were stained with 0.2% (w/v) Coomassie Brilliant Blue R250 in 50% methanol/10% acetic acid for 16 h. Destaining was carried out with 12.5% isopropanol/10% acetic acid. Alternatively, staining of the protein bands was performed using Simply Blue Safe Stain (Invitrogen) following the supplied protocol. Western blotting was performed according to the method of Towbin et al. [82]. Proteins were resolved by SDS-PAGE, transferred to a nitrocellulose membrane (Hybond-C Extra, GE Healthcare) and submitted to immunodetection using mouse IgG anti-His6 (Roche Applied Science, Germany) and goat anti-mouse IgG conjugated with horseradish peroxidase (Santa Cruz Biotechnolgy, Dallas, USA).

### N-terminal amino acid sequencing

N-terminal amino acid sequencing was carried out on a Shimadzu PPSQ-10 Automated Protein Sequencer (Kyoto, Japan). Protein samples were blotted onto a polyvinylidene fluoride (PVDF) membrane after SDS-PAGE and submitted to Edman degradation [83]. The phenylthiohydantoin (PTH) amino acids were detected at 269 nm after separation on a reversed phase C18 column (4.6 mm × 2.5 mm) under isocratic conditions, according to the manufacturer’s instructions.

### Effects of pH, temperature and metal ions on enzyme activity

To determine the effect of pH on enzyme stability, a solution of chitinase (150 ng/μL distilled water) was dialyzed for 1 h with continuous stirring against five buffer systems (each at 50 mM): glycine-HCl (pH 2.0 and 3.0), sodium acetate (pH 4.0 and 5.0), sodium phosphate (pH 6.0 and 7.0), Tris-HCl (pH 8.0) and glycine-NaOH (pH 9.0 and 10.0). The dialyzed samples were diluted 1:20 (v/v) with the respective buffers, and the residual chitinase activity was determined under standard assay conditions. The effect of temperature on enzyme stability was assessed by incubating the protein (150 ng/μL distilled water) at different temperatures (30, 40, 50, 60, 70, 80, 90 and 100°C) for 30 min in a water bath. After the heat treatment, the samples were stored at −20°C until the remaining chitinase activity was measured using standard assay conditions. For both factors, the relative activity was expressed as a percentage of the highest activity recorded at a certain pH or temperature, respectively. The effect of ions [Ba^2+^ (BaCl_4_), Ca^2+^ (CaCl_2_), Cu^2+^ (CuSO_4_), Fe^2+^ (FeSO_4_), K^+^ (KCl), Mg^2+^ (MgCl_2_), Mn^2+^ (MnCl_2_), NH_4_^+^ (NH_4_Cl), Ni^2+^ (NiCl) and Zn^2+^ (ZnSO_4_)], enzyme inhibitors (EDTA and β-mercaptoethanol) and a protein denaturant (SDS) on the enzymatic activity was investigated using *p*NP-(GlcNAc)_2_ and *p*NP-(GlcNAc)_3_ as substrates. The assay was carried out under standard conditions, including the ion (5 mM), or the inhibitor [EDTA (5 mM) or β-mercaptoethanol (5% v/v) or SDS (0.5, 1.0 and 2.0% w/v)] to be tested in the reaction mixture. The residual hydrolytic activity was determined and expressed as a percentage of the activity recorded in the absence of ions or enzyme inhibitors.

## Competing interests

The authors declare that they have no competing interests.

## Authors’ contributions

MDPL carried out the cloning experiments and the purification of the recombinant protein. MDPL, PGCL, PRC, TLB and SCM characterized the recombinant protein. FDAS and JTAO performed the chitinolytic activity assays. IMV determined the N-terminal sequence of the purified protein. HMP and TBG performed the comparative sequence analyses and homology modeling. MDPL prepared all of the figures. TBG conceived the work, coordinated the experiments and wrote the paper. All authors read and approved the final manuscript.

## Supplementary Material

Additional file 1: Figure S1 Production of *Cv*Chi45 in *E*. *coli*. Total chitinolytic activity was determined in the soluble cell lysates (A) and the cell-free medium (B) of induced *E*. *coli* BL21(DE3) cells harboring either the empty expression vector pET303/CT-His (●) or the recombinant vector pET-CV2935 (■) and cultivated in LB. Time 0 refers to the point (OD_600_ ≈ 0.4-0.5) at which IPTG was added (0.5 mM final concentration) to the cultures. Chitinolytic activity was measured as described in the Methods section, using colloidal chitin as a substrate. Click here for file

Additional file 2: Figure S2 Effect of cultivation medium on the soluble protein concentration and the chitinolytic activity of the protein secreted into the culture medium. Total chitinolytic activity (■) and soluble protein concentration (●) were determined in the cell-free culture medium of induced *E*. *coli* BL21(DE3) cells carrying the recombinant vector pET-CV2935 and cultivated in either LB (A) or TB (B). Time 0 refers to the point (OD_600_ ≈ 0.4-0.5) at which IPTG was added (0.5 mM final concentration) to the cultures. Protein concentration was determined using the Bradford method [80], and chitinolytic activity was measured as described in the Methods section, using colloidal chitin as a substrate. Click here for file

Additional file 3: Figure S3 Effect of ions on the hydrolytic activity of *Cv*Chi45. The hydrolytic activity of the recombinant chitinase was measured against the synthetic substrates *p*-nitrophenyl-β-D-*N*,*N*’-diacetylchitobiose [*p*NP-(GlcNAc)_2_] and *p*-nitrophenyl-β-D-*N*,*N*’-,*N*”-triacetylchitotriose [*p*NP-(GlcNAc)_3_] in the presence of different ions (5 mM), as described in the Methods section. In each treatment, the relative amount of enzymatic activity was expressed as a percentage of the hydrolytic activity recorded in the absence of ions (control). Click here for file

## References

[B1] Muzzarelli RAA Jollès P, Muzzarelli RAA Native, industrial, and fossil chitins Chitin and chitinases 1999 Basel: Birkhäuser

[B2] Hegedus D Erlandson M Gillott C Toprak U New insights into peritrophic matrix synthesis, architecture, and function Annu Rev Entomol 2009 54 285 302 10.1146/annurev.ento.54.110807.090559 19067633

[B3] Ubhayasekera W Structure and function of chitinases from glycoside hydrolase family 19 Polym Int 2011 60 890 896 10.1002/pi.3028

[B4] Henrissat B A classification of glycosyl hydrolases based on amino acid sequence similarities Biochem J 1991 280 Pt 2 309 316 1747104 10.1042/bj2800309 PMC1130547

[B5] Funkhouser JD Aronson NN Chitinase family GH18: evolutionary insights from the genomic history of a diverse protein family BMC Evol Biol 2007 7 96 10.1186/1471-2148-7-96 17594485 PMC1945033

[B6] Udaya Prakash NA Jayanthi M Sabarinathan R Kangueane P Mathew L Sekar K Evolution, homology conservation, and identification of unique sequence signatures in GH19 family chitinases J Mol Evol 2010 70 466 478 10.1007/s00239-010-9345-z 20480157

[B7] Iseli B Armand S Boller T Neuhaus JM Henrissat B Plant chitinases use two different hydrolytic mechanisms FEBS Lett 1996 382 186 188 10.1016/0014-5793(96)00174-3 8612749

[B8] Tews I Terwisscha Van Scheltinga AC Perrakis A Wilson KS Dijkstra BW Substrate-assisted catalysis unifies two families of chitinolytic enzymes J Am Chem Soc 1997 119 7954 7959 10.1021/ja970674i

[B9] Synstad B Gåseidnes S Van Aalten DMF Vriend G Nielsen JE Eijsink VGH Mutational and computational analysis of the role of conserved residues in the active site of a family 18 chitinase Eur J Biochem 2004 271 253 262 10.1046/j.1432-1033.2003.03923.x 14717693

[B10] Perrakis A Tews I Dauter Z Oppenheim AB Chet I Wilson KS Vorgias CE Crystal structure of a bacterial chitinase at 2.3 Å resolution Structure 1994 2 1169 1180 10.1016/S0969-2126(94)00119-7 7704527

[B11] Seidl V Chitinases of filamentous fungi: a large group of diverse proteins with multiple physiological functions Fungal Biol Rev 2008 22 36 42 10.1016/j.fbr.2008.03.002

[B12] Van Loon LC Rep M Pieterse CMJ Significance of inducible defense-related proteins in infected plants Annu Rev Phytopathol 2006 44 135 162 10.1146/annurev.phyto.44.070505.143425 16602946

[B13] Aam BB Heggset EB Norberg AL Sørlie M Vårum KM Eijsink VGH Production of chitooligosaccharides and their potential applications in medicine Mar Drugs 2010 8 1482 1517 10.3390/md8051482 20559485 PMC2885077

[B14] Chen J-K Shen C-R Liu C-L N-acetylglucosamine: production and applications Mar Drugs 2010 8 2493 2516 10.3390/md8092493 20948902 PMC2953398

[B15] Koburger JA May SO Isolation of Chromobacterium spp. from foods, soil, and water Appl Environ Microbiol 1982 44 1463 1465 7159087 10.1128/aem.44.6.1463-1465.1982 PMC242212

[B16] Vasconcelos ATR Almeida de DF Hungria M Guimarães CT Antônio RV Almeida FC Almeida LGP Almeida R Avles-Gomes JA Andrade EM Araripe J Araújo MFF Astolfi-Filho S Azevedo V Baptista AJ Bataus LAM Batista Da JS Beló A Van den Berg C Bogo M Bonatto S Bordignon J Brigido MM Brito CA Brocchi M Burity HA Camargo AA Cardoso D das D de P Carneiro NP Carraro DM The complete genome sequence of Chromobacterium violaceum reveals remarkable and exploitable bacterial adaptability Proc Natl Acad Sci U S A 2003 100 11660 11665 14500782 10.1073/pnas.1832124100 PMC208814

[B17] Grangeiro TB Jorge DM de M Bezerra WM Vasconcelos ATR Simpson AJG Transport genes of Chromobacterium violaceum: an overview Genet Mol Res 2004 3 117 133 15100993

[B18] Ikegami T Okada T Hashimoto M Seino S Watanabe T Shirakawa M Solution structure of the chitin-binding domain of Bacillus circulans WL-12 chitinase A1 J Biol Chem 2000 275 13654 13661 10.1074/jbc.275.18.13654 10788483

[B19] Hashimoto M Honda Y Nikaidou N Fukamizo T Watanabe T Site-directed mutagenesis of Asp280 suggests substrate-assisted catalysis of chitinase A1 from Bacillus circulans WL-12 J Biosci Bioeng 2000 89 100 102 10.1016/S1389-1723(00)90031-8 16232709

[B20] Ferrandon S Sterzenbach T Mersha FB Xu M-Q A single surface tryptophan in the chitin-binding domain from Bacillus circulans chitinase A1 plays a pivotal role in binding chitin and can be modified to create an elutable affinity tag Biochim Biophys Acta 2003 1621 31 40 10.1016/S0304-4165(03)00029-1 12667608

[B21] Hardt M Laine RA Mutation of active site residues in the chitin-binding domain ChBDChiA1 from chitinase A1 of Bacillus circulans alters substrate specificity: use of a green fluorescent protein binding assay Arch Biochem Biophys 2004 426 286 297 10.1016/j.abb.2004.03.017 15158679

[B22] Watanabe T Ito Y Yamada T Hashimoto M Sekine S Tanaka H The roles of the C-terminal domain and type III domains of chitinase A1 from Bacillus circulans WL-12 in chitin degradation J Bacteriol 1994 176 4465 4472 8045877 10.1128/jb.176.15.4465-4472.1994 PMC196264

[B23] Hashimoto M Ikegami T Seino S Ohuchi N Fukada H Sugiyama J Shirakawa M Watanabe T Expression and characterization of the chitin-binding domain of chitinase A1 from Bacillus circulans WL-12 J Bacteriol 2000 182 3045 3054 10.1128/JB.182.11.3045-3054.2000 10809681 PMC94488

[B24] Gilkes NR Henrissat B Kilburn DG Miller RC Warren RA Domains in microbial beta-1, 4-glycanases: sequence conservation, function, and enzyme families Microbiol Rev 1991 55 303 315 1886523 10.1128/mr.55.2.303-315.1991 PMC372816

[B25] Poon DKY Withers SG McIntosh LP Direct demonstration of the flexibility of the glycosylated proline-threonine linker in the Cellulomonas fimi Xylanase Cex through NMR spectroscopic analysis J Biol Chem 2007 282 2091 2100 17121820 10.1074/jbc.M609670200

[B26] Vaaje-Kolstad G Houston DR Rao FV Peter MG Synstad B Van Aalten DMF Eijsink VGH Structure of the D142N mutant of the family 18 chitinase ChiB from Serratia marcescens and its complex with allosamidin Biochim Biophys Acta 2004 1696 103 111 10.1016/j.bbapap.2003.09.014 14726210

[B27] Van Aalten DM Synstad B Brurberg MB Hough E Riise BW Eijsink VG Wierenga RK Structure of a two-domain chitotriosidase from Serratia marcescens at 1.9-Å resolution Proc Natl Acad Sci U S A 2000 97 5842 5847 10.1073/pnas.97.11.5842 10823940 PMC18521

[B28] Brameld KA Goddard WA The role of enzyme distortion in the single displacement mechanism of family 19 chitinases Proc Natl Acad Sci U S A 1998 95 4276 4281 10.1073/pnas.95.8.4276 9539727 PMC22479

[B29] Brameld KA Shrader WD Imperiali B Goddard WA Substrate assistance in the mechanism of family 18 chitinases: theoretical studies of potential intermediates and inhibitors J Mol Biol 1998 280 913 923 10.1006/jmbi.1998.1890 9671559

[B30] Van Aalten DM Komander D Synstad B Gåseidnes S Peter MG Eijsink VG Structural insights into the catalytic mechanism of a family 18 exo-chitinase Proc Natl Acad Sci U S A 2001 98 8979 8984 10.1073/pnas.151103798 11481469 PMC55359

[B31] Hsieh Y-C Wu Y-J Chiang T-Y Kuo C-Y Shrestha KL Chao C-F Huang Y-C Chuankhayan P Wu W-G Li Y-K Chen C-J Crystal structures of Bacillus cereus NCTU2 chitinase complexes with chitooligomers reveal novel substrate binding for catalysis: a chitinase without chitin binding and insertion domains J Biol Chem 2010 285 31603 31615 10.1074/jbc.M110.149310 20685646 PMC2951234

[B32] Li H Greene LH Sequence and structural analysis of the chitinase insertion domain reveals two conserved motifs involved in chitin-binding PLoS One 2010 5 e8654 10.1371/journal.pone.0008654 20084296 PMC2805709

[B33] Liu T Yan J Yang Q Comparative biochemistry of GH3, GH20 and GH84 β-N-acetyl-Dhexosaminidases and recent progress in selective inhibitor discovery Curr Drug Targets 2012 13 512 525 10.2174/138945012799499730 22280348

[B34] Keyhani NO Roseman S Physiological aspects of chitin catabolism in marine bacteria Biochim Biophys Acta 1999 1473 108 122 10.1016/S0304-4165(99)00172-5 10580132

[B35] Gooday G The ecology of chitin degradation Adv Microb Ecol 1990 11 387 430 10.1007/978-1-4684-7612-5_10

[B36] Streichsbier F Utilization of chitin as sole carbon and nitrogen source by Chromobacterium violaceum FEMS Microbiol Lett 1983 19 129 132 10.1111/j.1574-6968.1983.tb00525.x

[B37] Chernin LS Winson MK Thompson JM Haran S Bycroft BW Chet I Williams P Stewart GS Chitinolytic activity in Chromobacterium violaceum: substrate analysis and regulation by quorum sensing J Bacteriol 1998 180 4435 4441 9721280 10.1128/jb.180.17.4435-4441.1998 PMC107452

[B38] Sitrit Y Vorgias CE Chet I Oppenheim AB Cloning and primary structure of the chiA gene from Aeromonas caviae J Bacteriol 1995 177 4187 4189 7608101 10.1128/jb.177.14.4187-4189.1995 PMC177160

[B39] Chen JP Nagayama F Chang MC Cloning and expression of a chitinase gene from Aeromonas hydrophila in Escherichia coli Appl Environ Microbiol 1991 57 2426 2428 1768115 10.1128/aem.57.8.2426-2428.1991 PMC183590

[B40] Tsujibo H Orikoshi H Tanno H Fujimoto K Miyamoto K Imada C Okami Y Inamori Y Cloning, sequence, and expression of a chitinase gene from a marine bacterium, Altermonas sp. strain O-7 J Bacteriol 1993 175 176 181 8416892 10.1128/jb.175.1.176-181.1993 PMC196111

[B41] Wang SY Wu SJ Thottappilly G Locy RD Singh NK Molecular cloning and structural analysis of the gene encoding Bacillus cereus exochitinase Chi36 J Biosci Bioeng 2001 92 59 66 16233059 10.1263/jbb.92.59

[B42] Watanabe T Suzuki K Oyanagi W Ohnishi K Tanaka H Gene cloning of chitinase A1 from Bacillus circulans WL-12 revealed its evolutionary relationship to Serratia chitinase and to the type III homology units of fibronectin J Biol Chem 1990 265 15659 15665 2203782

[B43] Chernin LS De la Fuente L Sobolev V Haran S Vorgias CE Oppenheim AB Chet I Molecular cloning, structural analysis, and expression in Escherichia coli of a chitinase gene from Enterobacter agglomerans Appl Environ Microbiol 1997 63 834 839 9055404 10.1128/aem.63.3.834-839.1997 PMC168378

[B44] Gleave AP Taylor RK Morris BA Greenwood DR Cloning and sequencing of a gene encoding the 69-kDa extracellular chitinase of Janthinobacterium lividum FEMS Microbiol Lett 1995 131 279 288 10.1111/j.1574-6968.1995.tb07788.x 7557339

[B45] Jones JD Grady KL Suslow TV Bedbrook JR Isolation and characterization of genes encoding two chitinase enzymes from Serratia marcescens EMBO J 1986 5 467 473 16453672 10.1002/j.1460-2075.1986.tb04235.x PMC1166787

[B46] Robbins PW Albright C Benfield B Cloning and expression of a Streptomyces plicatus chitinase (chitinase-63) in Escherichia coli J Biol Chem 1988 263 443 447 3275646

[B47] Kadokura K Sakamoto Y Saito K Ikegami T Hirano T Hakamata W Oku T Nishio T Production and secretion of a recombinant Vibrio parahaemolyticus chitinase by Escherichia coli and its purification from the culture medium Biosci Biotechnol Biochem 2007 71 2848 2851 10.1271/bbb.70389 17986788

[B48] Cronin D MoenneLoccoz Y Dunne C OGara F Inhibition of egg hatch of the potato cyst nematode Globodera rostochiensis by chitinase-producing bacteria Eur J Plant Pathol 1997 103 433 440 10.1023/A:1008662729757

[B49] Park SK Lee M-C Harman GE The biocontrol activity of chromobacterium sp. strain C-61 against rhizoctonia solani depends on the productive ability of chitinase Plant Pathol J 2005 21 275 282

[B50] Wynn-Williams DD Distribution and characteristics of Chromobacterium in the maritime and sub-antarctic Polar Biol 1983 2 101 108 10.1007/BF00303175

[B51] Tata SJ Beintema JJ Balabaskaran S The lysozyme of Hevea brasiliensis latex - isolation, purification, enzyme kinetics and a partial amino acid sequence J Rubber Res Inst Malays 1983 31 35 48

[B52] Hoell IA Klemsdal SS Vaaje-Kolstad G Horn SJ Eijsink VGH Overexpression and characterization of a novel chitinase from Trichoderma atroviride strain P1 Biochim Biophys Acta 2005 1748 180 190 10.1016/j.bbapap.2005.01.002 15769595

[B53] Perkins SJ Johnson LN Machin PA Phillips DC Crystal structures of hen egg-white lysozyme complexes with gadolinium(III) and gadolinium(III)-N-acetyl-D-glucosamine Biochem J 1979 181 21 36 486153 10.1042/bj1810021 PMC1161121

[B54] De Vrije T Batenburg AM Jordi W De Kruijff B Inhibition of PhoE translocation across Escherichia coli inner-membrane vesicles by synthetic signal peptides suggests an important role of acidic phospholipids in protein translocation Eur J Biochem 1989 180 385 392 10.1111/j.1432-1033.1989.tb14660.x 2466667

[B55] Phoenix DA Kusters R Hikita C Mizushima S De Kruijff B OmpF-Lpp signal sequence mutants with varying charge hydrophobicity ratios provide evidence for a phosphatidylglycerol-signal sequence interaction during protein translocation across the Escherichia coli inner membrane J Biol Chem 1993 268 17069 17073 8349595

[B56] Puziss JW Fikes JD Bassford PJJ Analysis of mutational alterations in the hydrophilic segment of the maltose-binding protein signal peptide J Bacteriol 1989 171 2303 2311 2651397 10.1128/jb.171.5.2303-2311.1989 PMC209902

[B57] Akita M Sasaki S Matsuyama S Mizushima S SecA interacts with secretory proteins by recognizing the positive charge at the amino terminus of the signal peptide in Escherichia coli J Biol Chem 1990 265 8164 8169 2159471

[B58] Chou Y-T Gierasch LM The conformation of a signal peptide bound by Escherichia coli preprotein translocase SecA J Biol Chem 2005 280 32753 32760 10.1074/jbc.M507532200 16046390

[B59] Gelis I Bonvin AMJJ Keramisanou D Koukaki M Gouridis G Karamanou S Economou A Kalodimos CG Structural basis for signal-sequence recognition by the translocase motor SecA as determined by NMR Cell 2007 131 756 769 10.1016/j.cell.2007.09.039 18022369 PMC2170882

[B60] Musial-Siwek M Rusch SL Kendall DA Selective photoaffinity labeling identifies the signal peptide binding domain on SecA J Mol Biol 2007 365 637 648 10.1016/j.jmb.2006.10.027 17084862 PMC1851904

[B61] Von Heijne G Patterns of amino acids near signal-sequence cleavage sites Eur J Biochem 1983 133 17 21 10.1111/j.1432-1033.1983.tb07424.x 6852022

[B62] Kajava AV Zolov SN Kalinin AE Nesmeyanova MA The net charge of the first 18 residues of the mature sequence affects protein translocation across the cytoplasmic membrane of gram-negative bacteria J Bacteriol 2000 182 2163 2169 10.1128/JB.182.8.2163-2169.2000 10735858 PMC111264

[B63] Mergulhão FJM Summers DK Monteiro GA Recombinant protein secretion in Escherichia coli Biotechnol Adv 2005 23 177 202 10.1016/j.biotechadv.2004.11.003 15763404

[B64] Choi JH Lee SY Secretory and extracellular production of recombinant proteins using Escherichia coli Appl Microbiol Biotechnol 2004 64 625 635 10.1007/s00253-004-1559-9 14966662

[B65] Altschul SF Madden TL Schäffer AA Zhang J Zhang Z Miller W Lipman DJ Gapped BLAST and PSI-BLAST: a new generation of protein database search programs Nucleic Acids Res 1997 25 3389 3402 10.1093/nar/25.17.3389 9254694 PMC146917

[B66] Bendtsen JD Nielsen H Von Heijne G Brunak S Improved prediction of signal peptides: SignalP 3.0 J Mol Biol 2004 340 783 795 10.1016/j.jmb.2004.05.028 15223320

[B67] Gasteiger E Hoogland C Gattiker A Duvaud S Wilkins MR Appel RD Bairoch A Walker JM Protein Identification and Analysis Tools on the ExPASy Server The Proteomics Protocols Handbook 2005 New York: Humana Press 571 607

[B68] Marchler-Bauer A Zheng C Chitsaz F Derbyshire MK Geer LY Geer RC Gonzales NR Gwadz M Hurwitz DI Lanczycki CJ Lu F Lu S Marchler GH Song JS Thanki N Yamashita RA Zhang D Bryant SH CDD: conserved domains and protein three-dimensional structure Nucleic Acids Res 2013 41 D348 D352 10.1093/nar/gks1243 23197659 PMC3531192

[B69] Thompson JD Higgins DG Gibson TJ CLUSTAL W: improving the sensitivity of progressive multiple sequence alignment through sequence weighting, position-specific gap penalties and weight matrix choice Nucleic Acids Res 1994 22 4673 4680 10.1093/nar/22.22.4673 7984417 PMC308517

[B70] Holm L Rosenström P Dali server: conservation mapping in 3D Nucleic Acids Res 2010 38 W545 W549 10.1093/nar/gkq366 20457744 PMC2896194

[B71] Cantarel BL Coutinho PM Rancurel C Bernard T Lombard V Henrissat B The carbohydrate-active EnZymes database (CAZy): an expert resource for glycogenomics Nucleic Acids Res 2009 37 D233 D238 10.1093/nar/gkn663 18838391 PMC2686590

[B72] Warner SAJ Foster GD, Twell D Genomic DNA Isolation and Lambda Library Construction Plant Gene Isolation: principle and practice 1996 West Sussex: John Wiley & Sons 51 73

[B73] Sambrook J Fritsch E Maniatis T Molecular Cloning: A Laboratory Manual 1989 2 Cold Spring Harbor: Cold Spring Harbor Laboratory Press

[B74] Koshland D Botstein D Secretion of beta-lactamase requires the carboxy end of the protein Cell 1980 20 749 760 10.1016/0092-8674(80)90321-9 6448092

[B75] Boller T Gurr S, McPherson M, Bowles D Biochemical analysis of chitinase and β-1,3-glucanases Molecular Plant Pathology: A Practical Approach 1992 New York: Oxford University Press 23 30

[B76] Molano J Durán A Cabib E A rapid and sensitive assay for chitinase using tritiated chitin Anal Biochem 1977 83 648 656 10.1016/0003-2697(77)90069-0 603047

[B77] Boller T Gehri A Mauch F Vogeli U Chitinase in bean leaves: induction by ethylene, purification, properties, and possible function Planta 1983 157 22 31 10.1007/BF00394536 24263941

[B78] Reissig JL Storminger JL Leloir LF A modified colorimetric method for the estimation of N-acetylamino sugars J Biol Chem 1955 217 959 966 13271455

[B79] Frändberg E Schnürer J Evaluation of a chromogenic chito-oligosaccharide analogue, p-nitrophenyl-beta-D-N, N’-diacetylchitobiose, for the measurement of the chitinolytic activity of bacteria J Appl Bacteriol 1994 76 259 263 10.1111/j.1365-2672.1994.tb01625.x 8157546

[B80] Bradford MM A rapid and sensitive method for the quantitation of microgram quantities of protein utilizing the principle of protein-dye binding Anal Biochem 1976 72 248 254 10.1016/0003-2697(76)90527-3 942051

[B81] Laemmli UK Cleavage of structural proteins during the assembly of the head of bacteriophage T4 Nature 1970 227 680 685 10.1038/227680a0 5432063

[B82] Towbin H Staehelin T Gordon J Electrophoretic transfer of proteins from polyacrylamide gels to nitrocellulose sheets: procedure and some applications Proc Natl Acad Sci USA 1979 76 4350 4354 10.1073/pnas.76.9.4350 388439 PMC411572

[B83] Edman P Method for determination of the amino acid sequence in peptides Acta Chem Scand 1950 4 283 293

