# Supplementary figures and images for: Expression and efficient secretion of a functional chitinase from Chromobacterium violaceum in Escherichia coli
Source: BMC Biotechnol. 2013 Jun 1;13:46. doi: 10.1186/1472-6750-13-46 (PMC3701571; doi:10.1186/1472-6750-13-46)

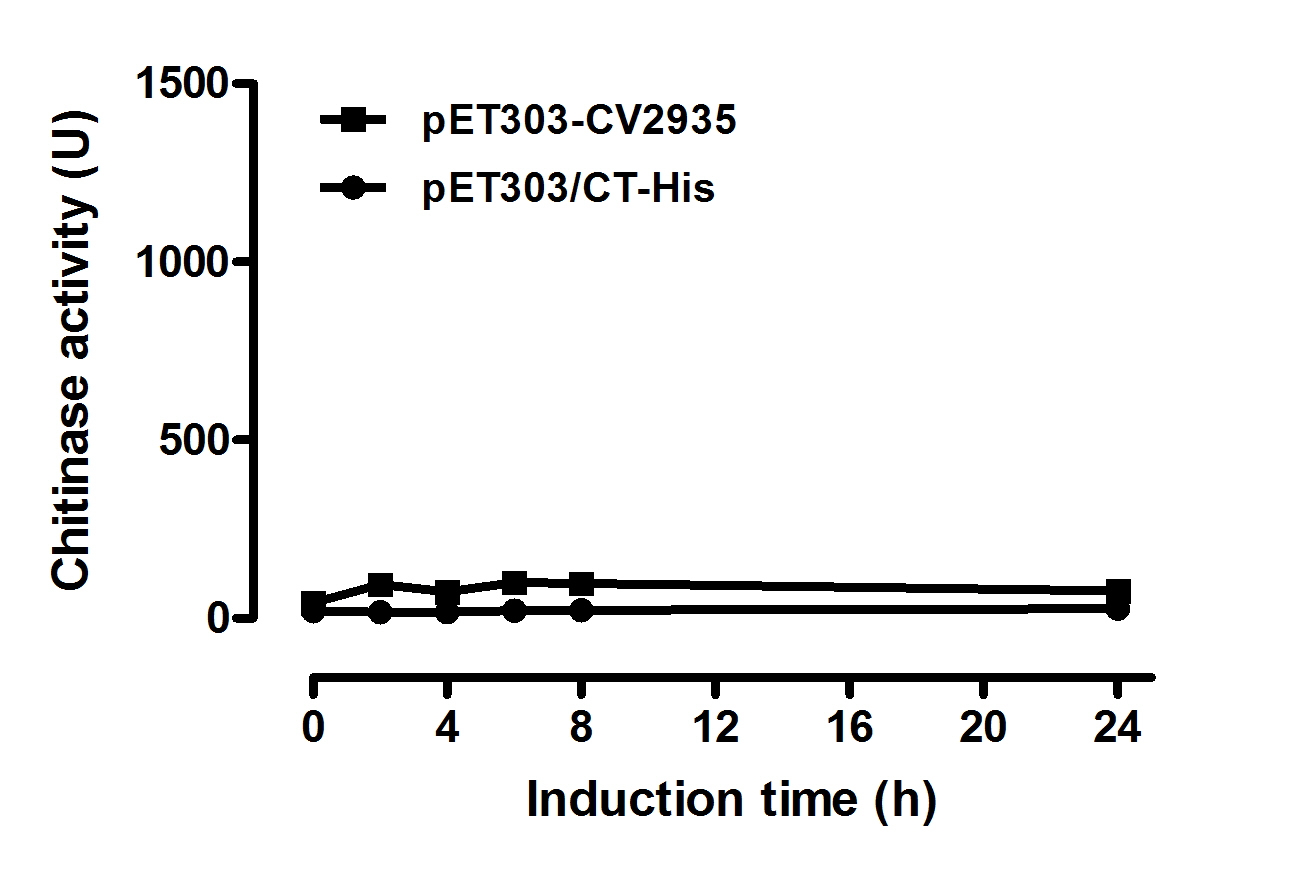


**A**


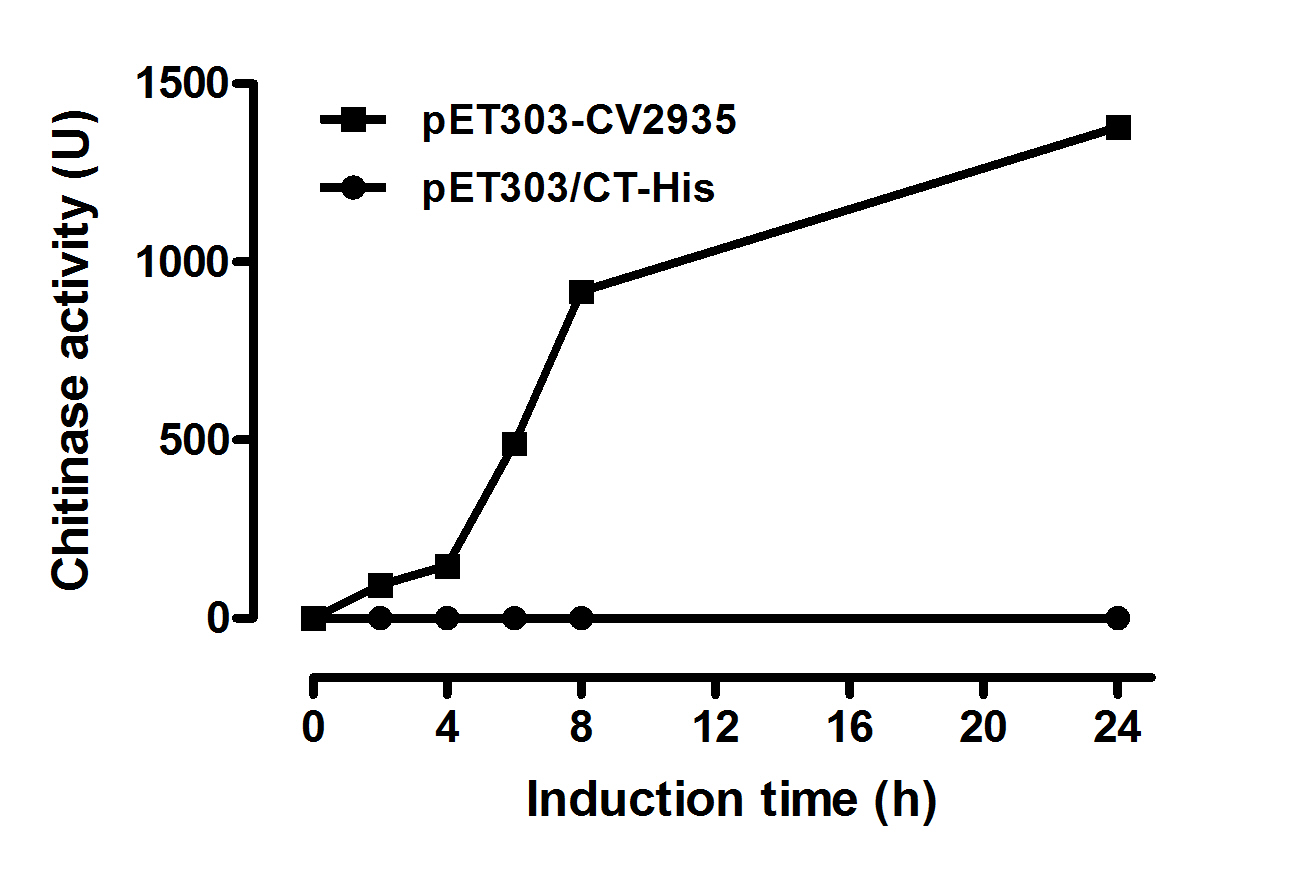


**B**

Supplement: Additional file 1: Figure S1 — Production of CvChi45 in E. coli. Total chitinolytic activity was determined in the soluble cell lysates (A) and the cell-free medium (B) of induced E. coli BL21(DE3) cells harboring either the empty expression vector pET303/CT-His (●) or the recombinant vector pET-CV2935 (■) and cultivated in LB. Time 0 refers to the point (OD600 ≈ 0.4-0.5) at which IPTG was added (0.5 mM final concentration) to the cultures. Chitinolytic activity was measured as described in the Methods section, using colloidal chitin as a substrate. [file 1472-6750-13-46-S1.doc]

**B**


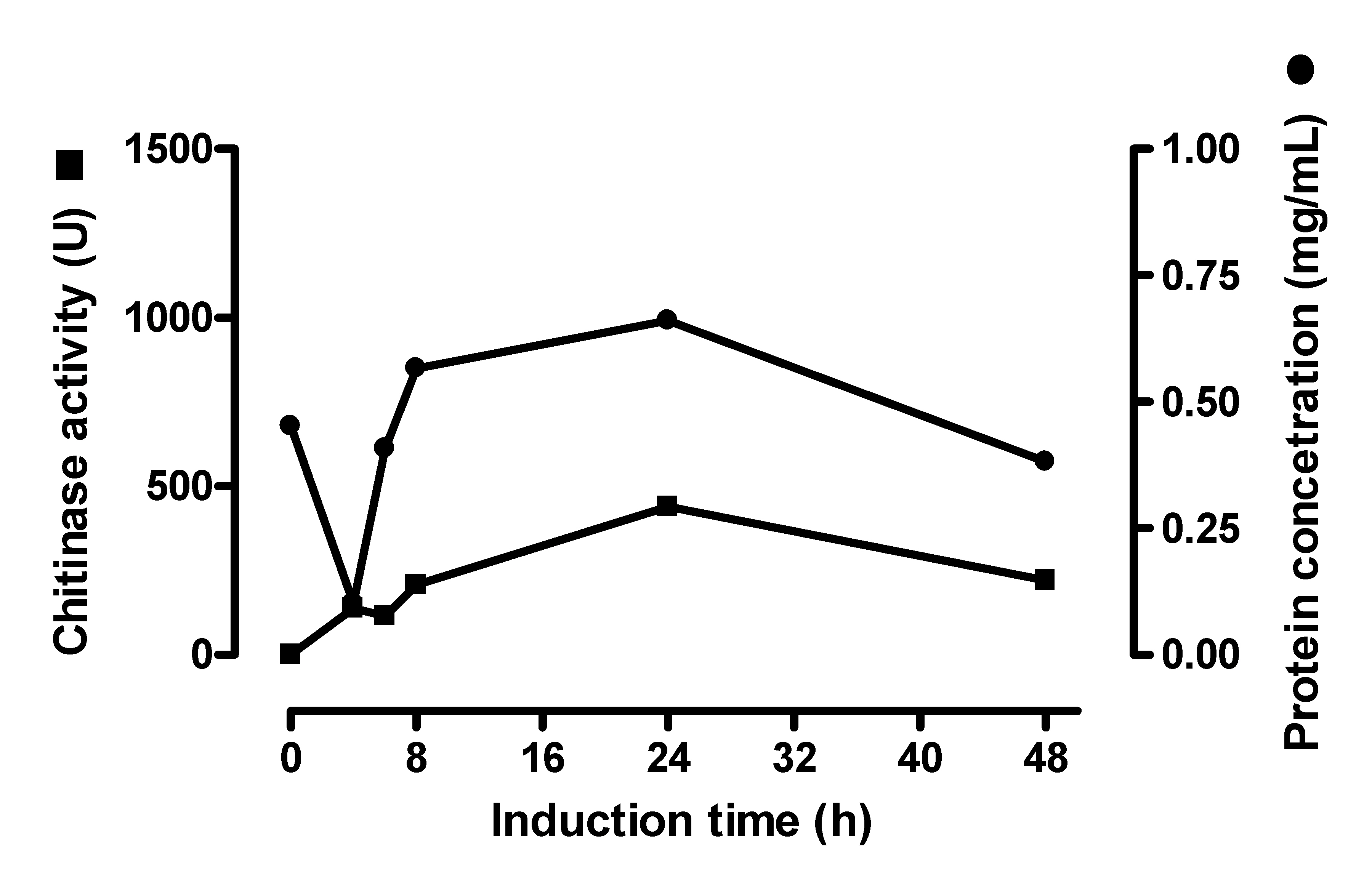


**A**


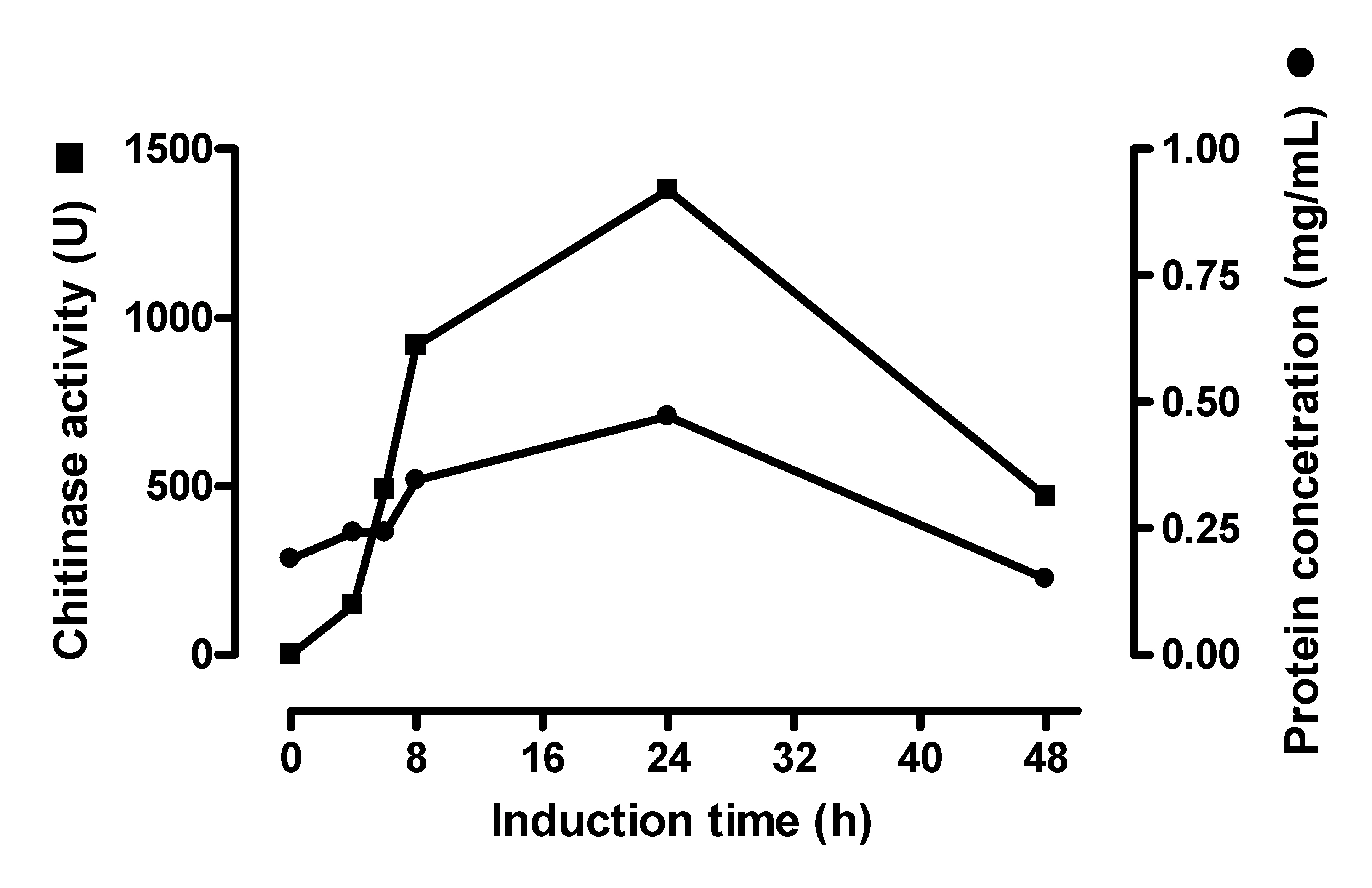

Supplement: Additional file 2: Figure S2 — Effect of cultivation medium on the soluble protein concentration and the chitinolytic activity of the protein secreted into the culture medium. Total chitinolytic activity (■) and soluble protein concentration (●) were determined in the cell-free culture medium of induced E. coli BL21(DE3) cells carrying the recombinant vector pET-CV2935 and cultivated in either LB (A) or TB (B). Time 0 refers to the point (OD600 ≈ 0.4-0.5) at which IPTG was added (0.5 mM final concentration) to the cultures. Protein concentration was determined using the Bradford method [80], and chitinolytic activity was measured as described in the Methods section, using colloidal chitin as a substrate. [file 1472-6750-13-46-S2.doc]

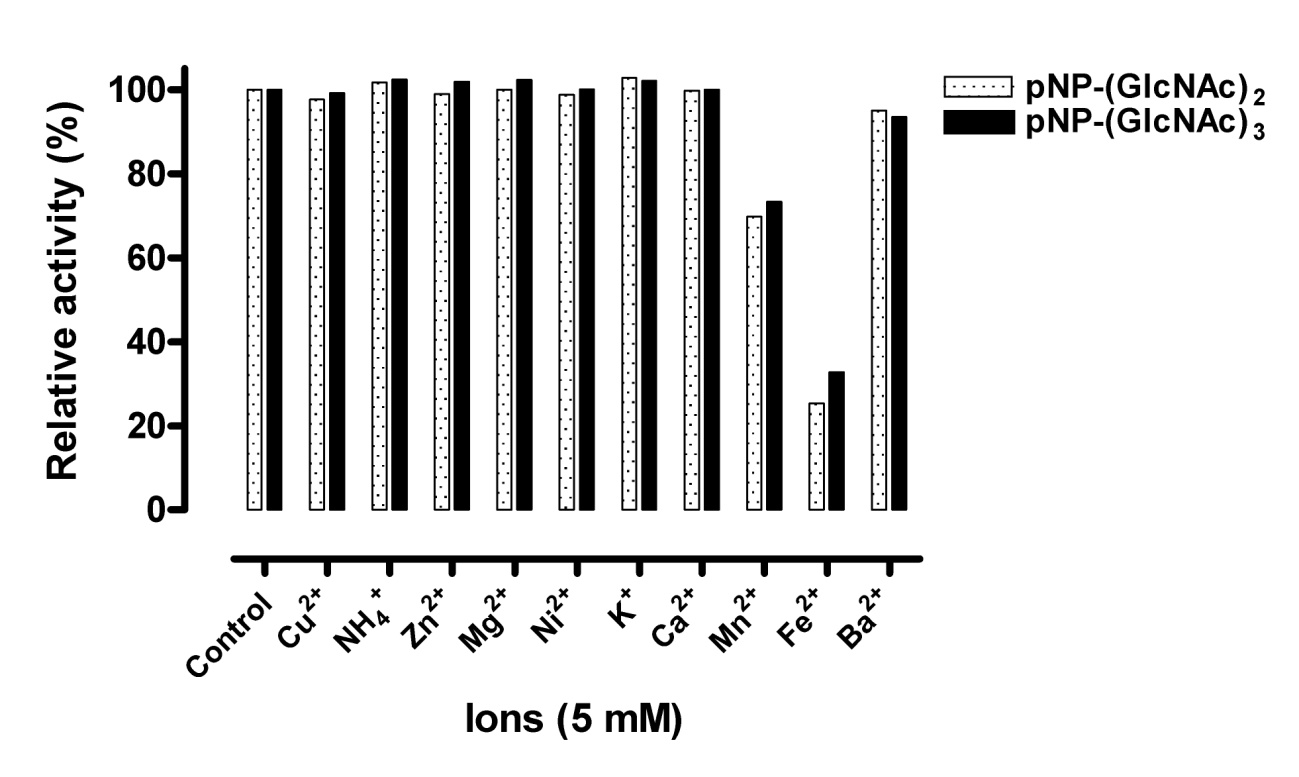

Supplement: Additional file 3: Figure S3 — Effect of ions on the hydrolytic activity of CvChi45. The hydrolytic activity of the recombinant chitinase was measured against the synthetic substrates p-nitrophenyl-β-D-N,N’-diacetylchitobiose [pNP-(GlcNAc)2] and p-nitrophenyl-β-D-N,N’-,N”-triacetylchitotriose [pNP-(GlcNAc)3] in the presence of different ions (5 mM), as described in the Methods section. In each treatment, the relative amount of enzymatic activity was expressed as a percentage of the hydrolytic activity recorded in the absence of ions (control). [file 1472-6750-13-46-S3.doc]
